# Comprehensive survey and evolutionary analysis of genome-wide miRNA genes from ten diploid *Oryza* species

**DOI:** 10.1186/s12864-017-4089-4

**Published:** 2017-09-11

**Authors:** Showkat Ahmad Ganie, Ananda Bhusan Debnath, Abubakar Mohammad Gumi, Tapan Kumar Mondal

**Affiliations:** Division of Genomic Resources, National Bureau of Plant Genetic Resources, Pusa, IARI Campus, New Delhi, 110012 India

**Keywords:** Cluster, Evolutionary rate, miRNA, *Oryza*, Selection, Transposons

## Abstract

**Background:**

MicroRNAs (miRNAs) are non-coding RNAs that play versatile roles in post-transcriptional gene regulation. Although much is known about their biogenesis, and gene regulation very little is known about their evolutionary relation among the closely related species.

**Result:**

All the orthologous miRNA genes of *Oryza sativa* (*japonica*) from 10 different *Oryza* species were identified, and the evolutionary changes among these genes were analysed. Significant differences in the expansion of miRNA gene families were observed across the *Oryza* species. Analysis of the nucleotide substitution rates indicated that the mature sequences show the least substitution rates among the different regions of miRNA genes, and also show a very much less substitution rates as compared to that of all protein-coding genes across the *Oryza* species. Evolution of miRNA genes was also found to be contributed by transposons. A non-neutral selection was observed at 80 different miRNA loci across *Oryza* species which were estimated to have lost ~87% of the sequence diversity during the domestication. The phylogenetic analysis revealed that *O. longistaminata* diverged first among the AA-genomes, whereas *O. brachyantha* and *O. punctata* appeared as the eminent out-groups. The miR1861 family organised into nine distinct compact clusters in the studied *Oryza* species except *O*. *brachyantha*. Further, the expression analysis showed that 11 salt-responsive miRNAs were differentially regulated between *O. coarctata* and *O. glaberrima.*

**Conclusion:**

Our study provides the evolutionary dynamics in the miRNA genes of 10 different *Oryza* species which will support more investigations about the structural and functional organization of miRNA genes of *Oryza* species.

**Electronic supplementary material:**

The online version of this article (10.1186/s12864-017-4089-4) contains supplementary material, which is available to authorized users.

## Background

MicroRNAs (miRNAs) are a class of non-protein-coding small RNAs playing versatile roles in post-transcriptional gene regulation. These regulatory genetic elements are formed from long self-complementary precursor sequences which in turn originate from still much longer primary miRNA sequences [1]. In contrast to the only seed sequence of mature miRNA for target recognition in animals, the plants employ entire mature miRNA sequence with near-perfect base pairing [2]. There are two main categories of the genes encoding the miRNA precursors [2]. In the first category, several highly expressed and greatly conserved miRNA gene families across plant lineages occur. miRNAs formed from such loci participate in various processes such as plant development and stress response. On the contrary, the second category contains less conserved miRNA genes that are expressed at lower levels. The roles of miRNAs belonging to this category are substantially unclear in plants [3]. Despite their small size, miRNAs have versatile functions. Through numerous experimental and genetic analyses, miRNAs have been shown to be involved in essential processes such as, plant growth and development, reproduction, and stress responses [4–9].

Some of the miRNA gene families in both plants and animals are highly conserved through millions of years [10, 11]. Nevertheless, specific and very recently evolved miRNA genes also occur in individual species [12, 13]. Gene duplication and subsequent sub- and neo-functionalization have been found leading to the expansion and specialization of highly conserved miRNA gene families [14, 15], whereas neutral evolution may act on the young and recently evolved miRNA genes [3]. Due to their evolutionary conservation, the miRNA genes with significant similarity can be found in different orthologous species [16]. This assumption is usually regarded as a basis for the identification of miRNA genes from the orthologous species. Several hypotheses regarding the origin of new miRNA genes exist such as, origin from the duplication of protein coding and miRNA genes [17–19], origin from terminal inverted repeats of transposable elements [13], and origin from random intronic or intergenic hairpin structures [20]. The plant miRNAs from the same family as well as their corresponding coding loci have been found not to be as diverse as the animal miRNAs, indicating that plant miRNA gene families have recently originated [21], and hence further assisting in the homology search of the miRNA genes in different plant species. However, the number of miRNA genes in plants has changed in a lineage-specific manner subsequent to the divergence of eudicots and monocots [22]. In addition, some members of plant miRNA precursors from the same family have also been found to be diverged in a lineage specific manner [23–25].

The comprehension about how genetic diversity influences the phenotypic differences among the species has been a major challenge in the evolutionary biology. The genus *Oryza*, due to its wealth of species, well-described phylogeny, plentiful genetic resources available and diversification across a wide-ranging ecology within a restricted evolutionary time period (~15 million years [MY]), has emerged as an exceptionally ideal model system for studying the short-term evolutionary dynamism shaping the plant kingdom [26–28]. The genus *Oryza* encompasses approximately 24 species that are classified into 10 distinctive genome types and distributed across a wide ecological range [26, 28]. It is widely established that wild species of *Oryza* have an immense genetic potential and agronomically important traits for the crop improvement. However, owing to the sterility barriers, majority of the *Oryza* species still remain underutilized for the rice improvement [29]. Genetic resources coupled with the enormous functional experimental resources of the AA-genomes of *Oryza* have certainly been attracting the attention of rice breeders. The interest of breeders in AA-genomes is also due to the fact that two species of this group are cultivated ones. Getting to the bottom of phylogenetic relationships among the different genomes and essentially the AA-genomes of *Oryza* will considerably pave the way for future efforts to explore and mine the beneficial alleles, which would eventually prove beneficial from the perspective of efficient rice germplasm conservation and utilization. Although the phylogenetic relationships among these genomes have been undertaken [30, 31], yet the realization of code and context of their genome evolution has not been achieved altogether. Through comparative genomic studies, it has been revealed that evolutionary dynamism among the different diploid genomes of *Oryza* is brought about by significant variations in the size of many protein-coding gene families which are in turn influenced by the directional selection [28]. Although few reports about the evolution of miRNA genes in rice exist [32–34], however, the evolution and selection among the *Oryza* genomes on the basis of non-protein-coding miRNA genes is still not well-characterized. Though a very recent report by Baldrich et al. [35] has identified and shown the conservation of some polycistronic miRNAs across the AA-genomes of *Oryza*, even their study has not identified all the orthologous miRNA genes of rice from the AA-, BB-, and FF-genomes of *Oryza*. The homology search is a reliable methodology for the identification of miRNA genes from the homologous species as well as genus, and has been exploited in the identification homologous miRNA gene sequences from different plant species. Therefore, in this study, we performed a comparative genome-based homology search to identify and analyze all the orthologous miRNA genes of rice in the 10 different *Oryza* species for uncovering the changes that might have occurred in them during the course of evolution. The 10 species were selected for this study on the basis of their sequenced genomes as well as due to immensely high importance of eight AA-genomes in studying the short evolutionary events for which the BB- and FF-genomes were selected as eminent out-group species. The changes in the number and sequence in different miRNA genes among the *Oryza* species will provide clues about the divergence and convergence of such genes. Further, the estimates of evolutionary divergence among the *Oryza* genomes from the genome-wide miRNA sequence variations would prove as a worthwhile effort beneficial to rice scientific community. This is the first report of its kind on the genome-wide investigation of all the orthologous miRNA genes of rice (*Oryza sativa subsp. japonica*) in the diploid *Oryza* group.

## Results and discussion

### Number and distribution of miRNA genes in the 10 Oryza genomes

Based on the sequence conservation of miRNA genes in plants, we identified an average number of ~326 miRNA genes (adopting proper stem-loop structures) by homology search in the 10 *Oryza* species. For numerous genes, more than one BLAST hits were found which were predicted to be the duplicated copies or paralogues of the corresponding miRNA genes. The numbers of miRNA genes and families were found to be considerably varied among the *Oryza* species (Table 1). For example, the number of miRNA gene families was found to be the least (28) in *O. brachyantha*, and the highest (184) in *O. rufipogon* (evolutionarily a close relative of *O. sativa*). In particular, this number was the least in *O. brachyantha* and *O. punctata*, and the gains in the number might have taken place during the course of evolution from FF- and BB- genomes to AA- *Oryza* genomes as well as during the domestication. This is fairly justified from the fact that progenators of *O. brachyantha* and *O. punctata* diverged from the domesticated rice *O. sativa* progenitor almost ~15 and 9.11 million years ago (MYA) respectively [36, 37]. It thus indicated that the expansion of the miRNA genes in AA- genomes has initiated in *O. meridionalis* and continued to a maximum in *O. nivara* and *O. sativa* (*indica*). These results suggest that the species-specific gain and loss of miRNA gene families in *Oryza* might have contributed to their evolution through morphological and reproductive divergence. However, as reported for *Drosophila* species [38], we used pre-miRNA sequences of *O. sativa* (*japonica*) (having almost 332 miRNA gene families and 592 genes as reported in miRBase) as queries for homology search. Therefore, it cannot be asserted with confidence that the *O. brachyantha* has the lowest number of miRNA genes among the studied genomes. It is possible that the miRNA gene sequences, specific only to *O. brachyantha* or other *Oryza* species in which reduced number of miRNA genes was observed, were not detected by the homology search using miRNA sequences from *O. sativa* (*japonica*). This possibility cannot be undermined as the experimental identification of miRNA genes from the *Oryza* species has not yet been accomplished completely. The different miRNA gene families in *Oryza* genomes were defined as per their corresponding orthologous gene families in *O. sativa* as reported in miRBase. It was found that the number of miRNA genes is exceedingly high as compared to the number of miRNA gene families (Table 1), signifying the importance of gene duplication for producing new miRNA genes. This was also reported for certain other plant species by some earlier studies [21, 22]. Each gene family was found to consist of one or many miRNA genes (average of 2.5 genes per gene family across the *Oryza*) and each miRNA gene possessed one or multiple (average of ~1.7 paralogues per gene) duplicated paralogous sequences. The average number of genes per gene family across *Oryza* is considerably higher than what has already been reported in animals [22, 39], suggesting that the average number of miRNA genes per gene family is higher in plants than animals. This finding indicates that the contributory role of gene duplication is higher in plants as compared to the animals for the generation of new miRNA genes [38]. This result also conforms to the finding that plants have smaller number of known miRNAs and their corresponding targets than animals; hence fewer but larger miRNA gene families occur in plants than animals [21]. In fact, it was found by homology search for numerous miRNA genes that an individual gene member of a particular miRNA family has more than one duplicated paralogues (Additional file 1), which are generally block duplicates as shown by their location mostly on different chromosomes. However, this sort of gene duplication in certain miRNA genes of *Oryza* is not reported for the corresponding orthologous miRNA genes of rice in miRBase which hence needs be investigated by further studies. Nevertheless, genomic duplication events cannot be taken into account as the sole causal agent for the expansion and divergence of the miRNA genes in the *Oryza* species, as also proposed for rice polycistronic miRNA genes [35]. We rule out the false identification of paralogues for a particular miRNA gene because we applied rigorous criteria for the same. The stem-loop structures of a conserved and a non-conserved miRNA from 10 *Oryza* species are given in Additional file 2.

**Table 1:** No. of different miRNA gene families derived from transposons, miRNA genes and paralogues in the 10 *Oryza* species

	No. of TE-derived miRNA gene families	Total no. of gene families	Total no. of genes	Total number of hits	Av. no. of genes/gene family	Av. no. of paralogous duplicates/gene
*O. barthi*	58	157	375	643	2.38	1.71
*O. brachyantha*	7	28	94	161	3.35	1.71
*O. glaberima*	52	143	339	682	2.37	2.01
*O. glumaepatula*	58	168	375	666	2.23	1.77
*O. longistaminata*	38	132	407	605	3.08	1.48
*O. meridionalis*	44	120	303	491	2.52	1.62
*O. nivara*	64	176	388	676	2.20	1.74
*O. punctata*	20	68	201	311	2.95	1.54
*O. rufipogon*	64	184	385	694	2.09	1.80
*O. sativa (indica)*	56	173	394	784	2.27	1.98

Analysis of genomic organisation of miRNA genes provided good information regarding their genomic locations and the rearrangements that might have occurred during the evolution. In all the *Oryza* species, miRNA genes were found broadly distributed throughout the corresponding genomes. For most of the miRNA genes in a particular species, the orthologues could be found in the other *Oryza* genomes (*O. brachyantha* and *O. punctata* being the strong exceptions in this case). However, the order of these orthologous genes on the corresponding chromosomes of *Oryza* genomes was either not found to be conserved or they were found on the different chromosomes of the *Oryza* species, indicating that the extensive rearrangements in miRNA genes might be due to several chromosomal inversions and translocations within or between the different chromosomes of *Oryza*. The highest number of homologous genes was found between *O. nivara* and *O. sativa* (*indica*) which is justified from the fact that they are evolutionarily separated by just 0.75 MY [40]. Analysis of the genomic organisation of miRNA genes revealed that, on average across *Oryza*, the maximum number (122) of miRNA genes are located in the intergenic regions (Additional file 3), whereas the minimum number (45) of them were found to be located at the boundaries of introns and exons (intron-exon). The similar results about the frequency of intergenic and intragenic miRNAs have also been found by certain mapping studies for other plant species [22, 41, 42]. It was also found that the number of different intergenic or intragenic miRNA genes was not uniform across *Oryza* species which further substantiates the idea that significant rearrangements in the genomes of *Oryza* have taken place during the course of evolution which might have caused the movement of miRNA genes from intergenic to intragenic locations and vice-versa.

### TE-derived miRNA genes in Oryza

Transposable elements are known to cause a plethora of changes in the gene expression and function of plants, which has significantly led to the understanding that TEs have played a fundamental part in the adaptive evolution of plant genomes [43]. The illustration of the relationships between transposable elements and miRNAs has been suggested to ease the elucidation of miRNA functionality [44]. To examine if any miRNA genes of *Oryza* species are derived from TEs, we employed the RepeatMasker (open-4.0.5) to screen the precursor sequences for the repetitive elements. We found that miRNA genes representing an average number of ~46 different miRNA gene families (averaging ~29% of total gene families across *Oryza*) from the studied *Oryza* species were found to possess the sequence similarity to the TEs (Table 1 and Additional file 1), implying that TEs might have given birth to these miRNA gene families. This result is corroborated by the findings of Zhang et al. [45], who also found a similar percentage (~29%) of TEs in the genomes of five *Oryza* species.. However, the result is in disagreement with the findings of Nozawa et al. [22], who found just 8% of miRNA gene families of different plant species to be derived from TEs. Among the different TE families found in the miRNA genes of *Oryza*, virtually none was found to be species-specific, indicating the importance of these transposons in the origin of miRNA gene families as well as in the evolution of *Oryza* genomes. Among the *Oryza* genomes, the maximum number of TE-derived miRNA gene families were found in *O. nivara* and *O. rufipogon*, whereas the minimum number of such gene families was found in the BB- and FF- genomes, indicating that expansion in the transposon number in miRNA genes from FF- and BB- to AA- genomes might have played a considerable contributory role in the evolution of *Oryza*. Although we found different TEs belonging to the families such as *copia*-, *gypsy*-like LTR retroelements, MITEs, MULEs, Harbinger etc. (Additional file 1) in the miRNA genes of *Oryza* species, the majority of the identified TEs were found to share similarity with nonautonomous DNA transposons known as MITEs. For instance, miR437, miR443, miR812, miR814, miR816, miR818, miR1862 and miR5788 families showed similarity to and hence might be derived from a MITE namely *STOWAWAY* which has been reported to be highly abundant in the rice genome [46]. Therefore, our results are consistent with the genomic enrichment of MITEs in rice [47]. Since the MITEs have been reported to play important roles in species diversity in rice [48], their relatively high abundance in the miRNA genes may also contribute to the species diversity in *Oryza*. Besides, many *gypsy*-like and *copia*-like LTR retrotransposons were also detected in the miRNA genes of *Oryza* with the *gypsy*-like elements being more abundant than *copia*-like elements. The higher abundance of *gypsy*-like elements than *copia*-like ones is also reported in rice genome [49]. For example, miR531 and miR5833 families were likely derived from the *copia*-like TEs, whereas miR2827, miR2907, miR5072, miR5074, miR5075 and miR5802 families appeared to have derived from the *gypsy*-like elements across the 10 studied *Oryza* genomes. Further, many miRNA gene families of *Oryza* were also found to be likely derived from TEs belonging to a MULE (*Mutator*-like elements) family known as *MuDR*. For example, we found that miR439, miR2122, miR2877, miR5149, miR5153 and miR5827 gene families originated from *MuDR* TEs virtually across the *Oryza*. This family of transposons has also been reported to be one of the abundant TE families in rice genome [50]. In addition, some more putative non-autonomous DNA transposons were also detected in the miRNA genes of the studied *Oryza* genomes (Additional file 1). Overall, these results indicate that the different families of transposable elements have distinct amplification patterns in the miRNA genes of *Oryza* species which might have contributed to some extent to the evolution of *Oryza* genomes.

### Evolutionary rates of miRNA genes in Oryza

**Fig. 1:**
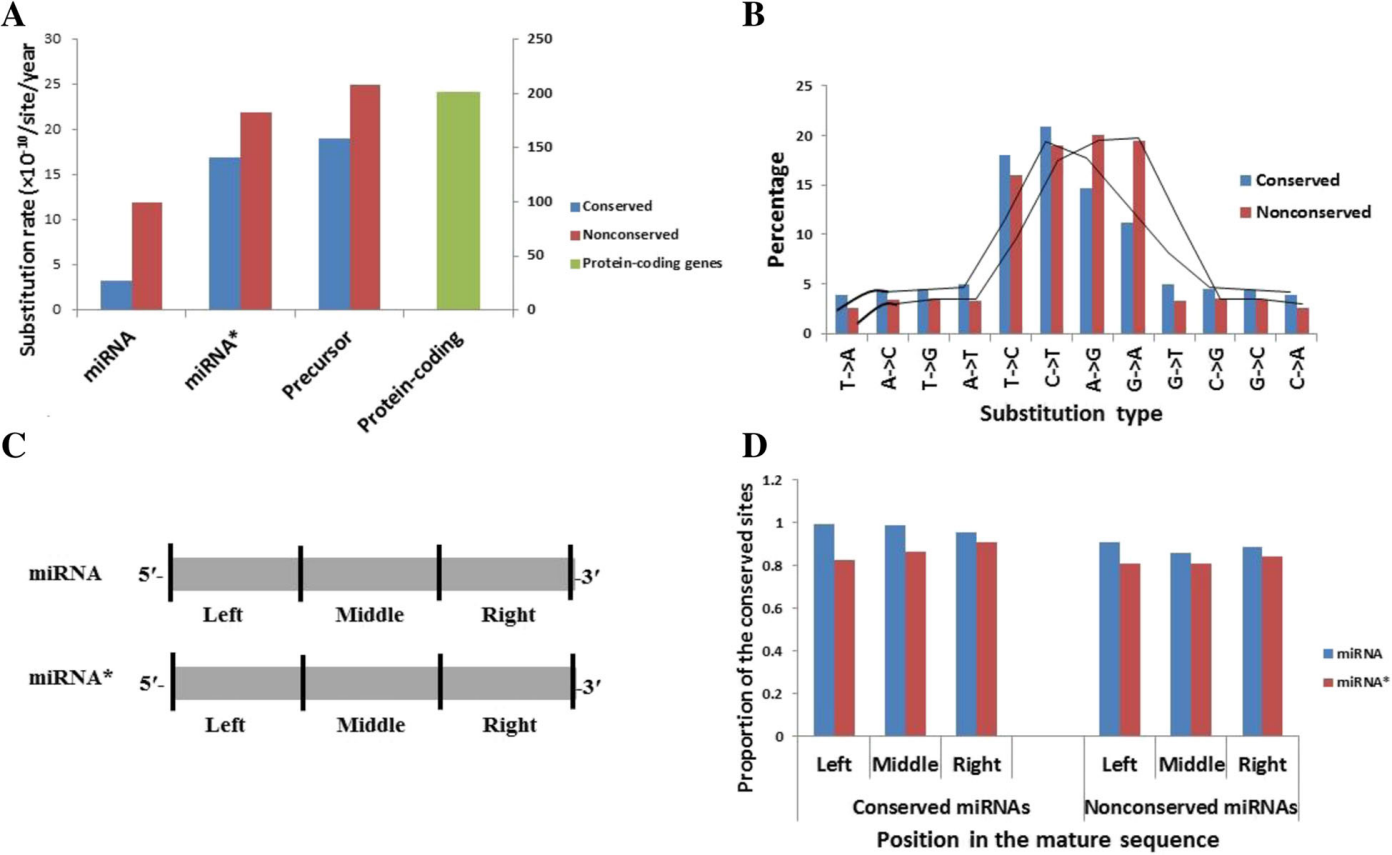
Evolutionary rate in the conserved and non-conserved miRNA genes of *Oryza.* Average substitution rates of different regions of miRNA genes and all the protein-coding genes of *Oryza.* All the regions of miRNA genes have much less substitution rates compared to protein-coding genes. The secondary axis depicts the substitution rate for protein-coding genes (**a**). Percentage of different nucleotide substitutions in the miRNA genes*.* T↔C transitions are more frequent in conserved miRNA precursors, whereas A↔G transitions occur more recurrently in non-conserved miRNAs (**b**). Schematic representation of three portions of an average sized 21 nt miRNA and miRNA* (**c**). Proportion of conserved sites in the different portions (left, middle and right portions) of mature and star regions of the miRNAs. Left portion (having the seed sequence) of miRNA and right portion (pairing with the left portion of miRNA) of miRNA* show higher conservation in both conserved as well as non-conserved miRNAs (**d**)

To investigate the evolutionary rates among the miRNA genes, the rates of nucleotide substitutions were estimated for different regions such as mature, star (complementary) and precursor sequences of the selected conserved and non-conservd miRNA genes of 10 *Oryza* genomes separately. It was found that all the analysed regions of conserved miRNA genes showed lesser substitution rates than their corresponding regions in the non-conserved miRNA genes with the mature region exhibiting the highest difference in the substitution rate between the two groups of miRNA genes (Fig. 1a and Additional file 4). Sequences in the stem region, from where the mature sequence is derived, were however found to be more conserved than other regions in both conserved and non-conserved pre-miRNAs of *Oryza* species (Additional file 5). The rates of substitution in mature regions of conserved and non-conserved miRNA genes were found to be ~3.2 × 10^−10^/site/year and 12 × 10^−10^/site/year respectively, which are much lesser than their corresponding star/complementary counterparts and precursors (Fig. 1a). The slower rates of substitution in mature region than star and precursor sequences are legitimate owing to the fact that only the mature sequences are essential to identify the targets for regulating thegene expression, and hence are under the strong selection pressure. However, when compared with the substitution rates in the protein-coding genes (*Ka/Ks* ratio of all orthologous protein-coding genes of *Oryza* given in Additional file 6), even the star and precursor sequences of both conserved as well as non-conserved miRNA genes exhibited considerably lower substitution rates, implicating that star and precursor sequences also undergo purifying selection albeit lesser than mature region. Similar results have also been found in some earlier studies [22, 38, 51]. To know if the nucleotide substitutions in the precursor sequences of the selected conserved and non-conserved miRNA genes favour transitions or transversions, the transition/transversion ratios were calculated. The results showed that in a given precursor alignment involving all the *Oryza* species, transitions were more frequent (ratio > 0.5) than transversions (Additional files 4 and 7). Higher ratio was found in the non-conserved precursors than conserved pre-miRNAs. For conserved pre-miRNAs, the most recurrent substitutions (averaged for all precursors) were C-to-T (20.85%) followed by T-to-C (18.03%) and A-to-G (14.61%), whereas for non-conserved pre-miRNAs the most frequent transitions were A-to-G (20.10%) followed by G-to-A (19.43%), C-to-T (18.95%) and T-to-C (15.98%) (Fig. 1b and Additional file 8). Hence, T↔C transition are more frequent in conserved precursors than non-conserved ones and vice-versa for A↔G transitions indicating that differential frequency of transition substitutions in pre-miRNAs may act as a crucial factor in determining the conservation of precursors in *Oryza*. These results are substantiated by the similar results obtained for rice pre-miRNAs in previous studies [52, 53]. Since it has been demonstrated that true SNPs are amply biased to transitions than transversions [52], the different transition substitutions found in the pre-miRNAs of different *Oryza* species may prove as the potential SNPs to be studied in further studies. This transition bias may signify the higher level of methylation in the pre-miRNAs in the *Oryza* genomes [52] with the higher level in non-conserved than conserved precursors.

To investigate the sequence conservation in the different regions (left, middle and right regions) of mature and star sequences of the selected conserved and non-conserved miRNA genes, the corresponding mature and star sequencesfrom the 10 *Oryza* species were aligned seperately. It was found that the left region (at 5′-end) of both conserved and non-conserved mature miRNAs showed the highest conservation than the middle and right (at 3′-end) regions (Fig. 1c and Additional file 9). This might be due to the presence of the seed-like sequence (2nd to 7th nt) in this region of mature miRNAs. This result implies that this conserved seed-like region is particularly under a strong selection pressure across the *Oryza* species, indicating the importance of this region in target recognition than the middle and right regions, which is substantiated by some earlier studies [54, 55]. In the conserved miRNA genes, the right region of the mature miRNAs was found to be the least conserved, whereas the middle region of the mature miRNAs in non-conserved miRNA genes was found to be the least conserved. In case of star sequences of conserved miRNA genes, the proportion of conserved sites in different regions was found to be reverse as that of their corresponding mature sequences, whereas in non-conserved miRNA genes, the star regions had the similar proportion of conserved sites in the left and middle regions but slightly higher in the right region. Consequently, the proportion of unpaired sites in conserved miRNA-miRNA* duplexes might be higher in the right region, whereas in non-conserved miRNAs the higher number of unpaired sites might prevail at the middle of miRNA-miRNA* duplexes. To a certain degree these findings are in agreement with the results obtained for different plant species [22], however, some discrepancy may be due to the analysis of different miRNA genes and the different plant species representing various taxonomical positions.

Further, to know if there was a relation between the base content of miRNA genes and their conservation, we calculated the base composition (AT- and GC-content) of mature, star and precursor regions of the selected conserved as well as non-conserved miRNA genes of *Oryza*. It was found that AT-content was lesser than GC-content in mature and star regions of both conserved as well as non-conserved miRNA genes (Additional files 10 and 11). However, as also reported for rice miRNA genes [56], AT-content in the conserved *Oryza* pre-miRNAs was lesser than GC-content as compared to the non-conserved miRNAs which possessed relatively higher AT-content than GC-content, indicating that diverged forces might link base content and conservation in miRNAs.

### Selection and patterns of sequence variation

**Fig. 2:**
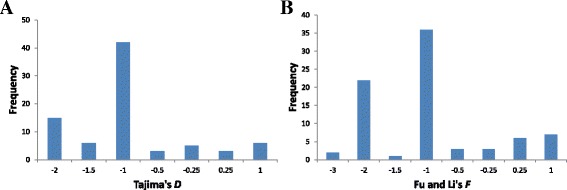
Distribution of Tajima’s *D* (**a**) and Fu and Li’s *F* values (**b**) for 80 miRNA loci in 10 *Oryza* species. Negative values for both the tests are shown by most of the loci signifying a recent selective sweep in *Oryza.* The values on the X-axis are not drawn to the scale

To estimate the neutrality of sequence polymorphisms for the miRNA genes of *Oryza*, Tajima’s *D* [57] and Fu and Li’s *F* [58] were performed on 80 different miRNA loci. Both of the tests are widely used as neutrality tests because they can detect both positive and balancing selections as well as provide information about a skew in the spectrum of allele frequency [59, 60]. Under neutral equilibrium, the mean Tajima’s *D* for the sequence variants in a population is expected to be zero, whereas the positive selection is inferred from negative values of Tajima’s *D* [33]. The values of Tajima’s *D* as well as Fu and Li’s *F* were calculated for each of the 80 miRNA loci (pre-miRNAs) from the 10 different *Oryza* species. A distinct pattern of distribution of values for both the tests was observed (Fig. 2a and b, Additional file 12). Several loci across the *Oryza* were found to be non-neutral sequence variants with significant probabilities. Similar distinct patterns of Tajima’s *D* values were also observed in the different miRNA loci of domesticated rice and *Arabidopsis* [33, 60]. Most of the loci showed the negative values for both the tests, however, only 11 miRNA loci were found to exhibit significant negative values for both the tests across the ten *Oryza* genomes (*p* < 0.05). Among the pre-miRNAs examined across *Oryza*, pre-miR172d put the most negatively significant Tajima’s *D* value (−2.08) on display and hence the departure from the neutral expectation was marginal at this locus. Consistent with the Tajima’s test, Fu and Li’s test also resulted in a significantly negative value at this locus (−2.67). The negative values of one or both of the tests for many of these loci are consistent with some earlier studies on rice and Arabidopsis [33, 34, 61]. However, many loci showing the negative values across *Oryza* species for one or both the tests were contrastingly found by some earlier studies to exhibit the significantly positive values. For example, miR399a loci from the different accessions of *O. rufipogon* showed positive value (1.21) for Tajima’s *D* [33]. Another locus (miR393a), analysed across the different accessions of Arabidopsis, has been reported to possess a significantly positive Tajima’s *D* value (3.39). On the other hand, the patterns of sequence diversity at some pre-miRNA loci showed a clear-cut departure from the neutrality owing to the highly positive but non-significant values for both the tests (e.g.pre-miR156k, pre-miR159a, pre-miR529b and pre-miR2093). However, several loci displaying positive values for one or both the neutrality tests, as found in the present study, have been found by some studies to possibly undergo a selective sweep. For example, miR172a locus was found to undergo positive selection [61]. Such a difference in the neutrality tests at these specific loci must owe to the differences in plant species studied in each report. It hence verifies that nature has targeted the miRNAs as the effective elements for the differential accumulation of variations in populations under selection pressures [33]. The nucleotide diversity among the studied *Oryza* species varied from 0.0028 at pre-miR1425 locus to 0.2562 at pre-miR1320 locus. The average sequence diversity (*π*) at 11 miRNA loci (with the significantly negative values for both the neutrality tests) in cultivated rice was estimated to be only ~13% of the sequence diversity in wild species of *Oryza* (Additional files 13 and 14). In other words, it implied that these miRNA loci lost ~87% of the average sequence diversity during the course of domestication and hence might probably be associated with the process of domestication. Therefore, these results strongly advocate, and are consistent with the fact that wild species of rice have an extensive and rich hidden genetic potential that is significantly untapped for the crop improvement programmes. Similar losses of sequence diversity at the different miRNA loci in cultivated rice than that of their wild progenitors have also been recorded earlier [32, 34]. The results obtained here also implicate that the process of selection in *Oryza* might lie behind the sequence variations/polymorphisms at the corresponding miRNA loci.

### Phylogenetic analysis of conserved and non-conserved miRNAs from the Oryza species

**Fig. 3:**
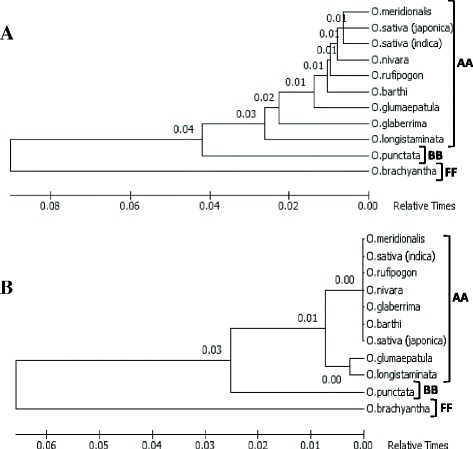
Phylogenetic relationships among the AA-, BB- and FF-genomes of *Oryza* based on the precursor sequences of non-conserved-miR812a (**a**) and conserved-miR156l (**b**). The evolutionary history was inferred with the Neighbor-Joining method using MEGA6. Both the tree topologies depict BB- and FF-genomes as the eminent outgroups, whereas *O. longistaminata* can be seen as the earliest divergent species among the AA-genomes

In plants, the compact series of rather recent events of speciation are typically exemplified by the *Oryza* species with AA-diploid genomes [37]. However, it is not well-known when and how these species diversified and therefore has been an issue of profound interest that is yet to be resolved. Therefore, a phylogenetic analysis of all the eight AA-diploid genome species along with the other two diploid species *O. punctata* and *O. brachyantha* (with BB- and FF- genomes respectively) was performed based on the sequences of miR156l and miR812a which represent conserved and non-conserved groups of miRNA genes respectively. The precursor sequences of these two miRNA genes were selected for the phylogenetic analysis, as the precursor sequences are important in understanding the evolutionary relationship of miRNA genes [25]. The topologies of both the dendrograms generated from the two miRNAs almost precisely harmonized with the evolution of *Oryza* genomes. From the dendrograms generated from the both conserved as well as non-conserved miRNA precursor sequences (Fig. 3a and b), it can be seen that *O. longistaminata*, native to most of the sub-Saharan Africa and Madagaskar, is the earliest divergent species among the AA-genomes. The markedly early divergence of *O. longistaminata* from the other AA-genomes of *Oryza* was also substantiated by some earlier investigations [40, 62]. Further, in both the dendrograms, *O. brachyantha* and *O. punctata*, were found to be the eminent out-groups to be distinguished phylogenetically from the *Oryza* genomes in AA-group. The AA genomes diverged from FF progenitors almost ~15 MYA [61], whereas the progenitors of AA- and BB- genomes diverged 9.11 MYA during the Miocene epoch [40]. The BB- and FF-genomes have also been reported by some earlier studies to act as the out-groups from the AA-genomes in other phylogenetic relationships [28, 37, 63]. Once diverged from the BB-genome species, AA-genomes radiated as a separate cluster, bringing the whole diversified AA-genome lineage into existence with a very recent evolutionary history of 2.93 MY [37]. It thus suggested that miRNAs can act as pivotal genetic elements for the analysis and inference of phylogenetic relationships among the different *Oryza* genomes, as abundant architectural complexities and dynamic changes in these regulatory modules must have occurred in *Oryza* during their 15 MY of evolutionary history. Comprehensions about the evolution of *Oryza* genomes is very important from the perspective of agronomically important traits because the diversification in the *Oryza* lineage is mainly accredited to the untapped reservoir of genome changes capitalizing which would eventually prove beneficial for the improvement of rice and other crops [63].

### Genomic organization and conservation of miR1861 family in Oryza

**Fig. 4:**
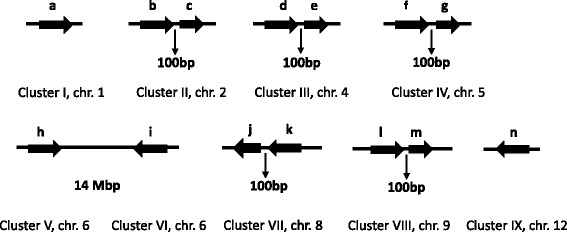
Genomic organization of the rice osa-miR1861 gene family as the nine distinct clusters on eight different chromosomes. Clusters point towards right direction if they are on the (+)- strand and vice-versa for (−)- strand of DNA. The 2nd member of a 2-membered cluster is depicted as shorter than the first owing to the 22-nt deletion in the 2nd one. Numbers below the 2-membered clusters represent the intergenic distance between the 2 genes

Among the different non-conserved miRNA gene families of rice, miR1861 family is unique in that it is organized into distinct compact clusters that can be transcribed as single units. This miRNA family regulates the growth and development of rice grains [64]. It has also been identified playing important roles in response to drought stress at the vegetative stage of rice [65]. We attempted to study the organization and sequence variation of this miRNA gene family among the 10 different *Oryza* species. Firstly, in rice, we found that this gene family has 14 members which have very similar sequences as already discovered [64]. These members were found to localize as nine clusters in rice genome among which clusters-II, III, IV, VII and VIII possess two tandem miRNA members each, whereas clusters-I, V, VI and IX have one member each (Fig. 4). In order to extract these clusters from the 10 *Oryza* species, BLAST search was performed in gramene against each species with the miR1861-clusters from rice as queries. All miR1861-clusters could be retrieved from the genomes of the *Oryza* species except *O. brachyantha*, which could be due to the high level of divergence of *O. brachyantha* from the rice (*Oryza sativa subsp. japonica*). Cluster on a particular chromosome from one species was found to lay on the same or mostly on the different chromosomes in other *Oryza* species, indicating that substantial shifts in the different genomic regions have taken place during the course of evolution in *Oryza*.

**Fig. 5:**
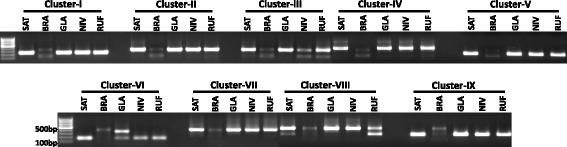
Mining of nine miR1861-clusters from the different species of *Oryza* genus. No cluster amplified from *O. brachyantha* at these nine loci. On the extreme left is the 100 bp marker. SAT-*O. sativa* (*indica*), BRA-*O. brachyantha*, GLA-*O. glaberrima*, NIV-*O. nivara*, RUF-*O. rufipogon*

To validate the presence of such clusters in *Oryza* genomes, primers specific to individual clusters were designed and used to mine these clusters from *O. sativa indica*, *O. brachyantha* (as negative control because no miRNA gene belonging to the miR1861 family could be identified through our *in-silico* analysis in this species)*, O. glaberrima, O. rufipogon* and *O. nivara* (Fig. 5). In harmony with the BLAST results of this study, we validated that no cluster could be amplified from the *O. brachyantha*, whereas all the clusters were found to be present in the rest four species (Fig. 5). The lesser number of miRNA genes and the absence of miR1861 clusters in *O. brachyantha* might be reasoned out to the finding that illegitimate and unequal homologous recombination in this species have resulted in the elimination of ancient gene families massively which gave rise to its smaller genome size than rice [66]. The sequenced clusters from the four *Oryza* species as well the clusters of other five studied *Oryza* species (retrieved from gramene) were aligned using clustalW to analyse the possible structure and sequence variations. Multiple sequence alignment of all the clusters individually from all the species revealed a significant conservation in the cluster sequences, although the presence of distinct nucleotide substitutions and indels were also evident (Additional file 15). Cluster-VII was found to be the most diverged among the *Oryza* species, indicating that the miR1861j and miR1861k constituting this cluster might have diverged among the species during the evolution and hence possibly might have acquired different roles. The presence of high sequence conservation in the *Oryza* clusters could possibly be attributed to the very less divergence times among the *Oryza* species [37]. The high sequence conservation among the members of a particular two-membered cluster of this miRNA gene family might point towards their origin from the same ancestral copy and hence must be the outcomes of rather recent tandem duplication events. As found in rice [64], precursors in each two-membered cluster of 10 *Oryza* species were also found to be bridged by a sequence of ~100 nt (Additional file 16 A). Despite the significant sequence conservation, the genomic configuration of miR1861 clusters was found not to be conserved due to the random scattering of clusters across the *Oryza* genomes. The deletion of 22 nt immediately downstream of the mature sequence in the second precursor (as found by Zhu et al. [64] in osa-miR1861 family of rice) of all the two-membered miR1861 clusters was consistently present in all the *Oryza* species (Additional file 16 B), suggesting that this deletion in the two-membered clusters was under the strong purifying selection. It was also inferred that the original pre-miRNA in all the species might have had undergone a tandem duplication followed by the deletion of 22 nt in the second precursor or the 22-nt insertion in the first precursor forming a two-membered cluster. The subsequent segmental duplication after this duplication generated the other tandemly configured members of this family [64]. Hence, these duplication events once occurred in the ancestor were conserved across the *Oryza*. The recent divergence in the *Oryza* is hence proven from the conservation in the sequence and structure of the miR1861 family members.

**Fig. 6:**
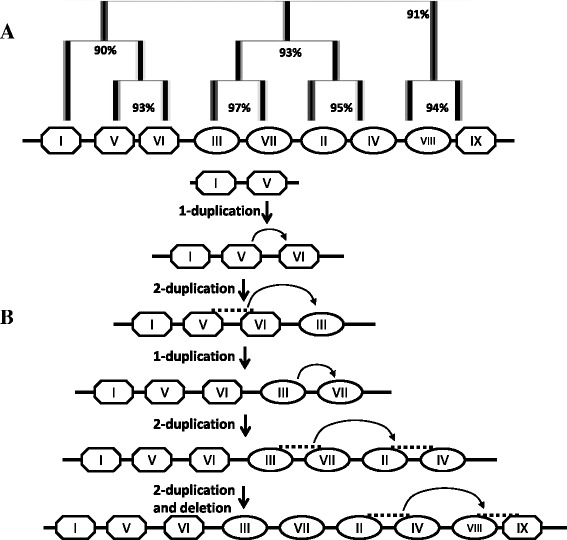
A probable evolutionary history of nine osa-miR1861 clusters in rice. Relationship among the nine clusters of osa-miR1861 family based on their sequence similarity as depicted by percentage numbers (**a**). A possible evolutionary path for nine clusters of osa-miR1861 family (**b**). Octagons and ovals represent the single gene and 2-gene clusters respectively. Arrows show the direction of possible duplication. Duplicated clusters are indicated by the dashed line. The bold line on which the clusters are drawn inaccurately symbolise the different regions of rice genome rather than a chromosome

To study the relationship among the miR1861 clusters of rice, multiple sequence alignment was performed among them. As shown in the Additional file 17, the similarity among the nine clusters was very high (at least 83% similarity), implying that the clusters were derived from rather recent segmental duplication events. The similarity between duplicated pairs of clusters was found to be even higher (>90%), evidencing duplication as the possible source of their origination. As per the similarity among the osa-miRNA1861 clusters, an apparent evolutionary pathway of the nine clusters was developed. Since the origin of the ancestral osa-miR1861 gene is not well-defined, we assumed on the basis of phylogenetic tree (Fig. 6a) that osa-miR1861a and osa-miR1861h (cluster I and V) pre-existed in the ancestor of cultivated rice. From the evolutionary history (Fig. 6b), it can be assumed that 1-duplication (duplication of a single gene) event in the cluster V resulted in the formation of cluster VI. A subsequent 2-duplication (duplication of two genes at a time) event in clusters V and VI gave rise to the 2-membered cluster III, from which another 2-membered cluster VII was formed after a 1-duplication event. This was followed by 2-duplication event in the 2-membered clusters III and VII producing yet another two 2-membered clusters II and IV. The osa-miR1861 family was put into its current configuration by a final 2-duplication event followed a likely deletion in the clusters II and IV, consequently bringing about a 2-membered (Cluster VIII) and a 1-membered (cluster IX) cluster.

### Expression analysis of salt-responsive miRNAs

**Fig. 7:**
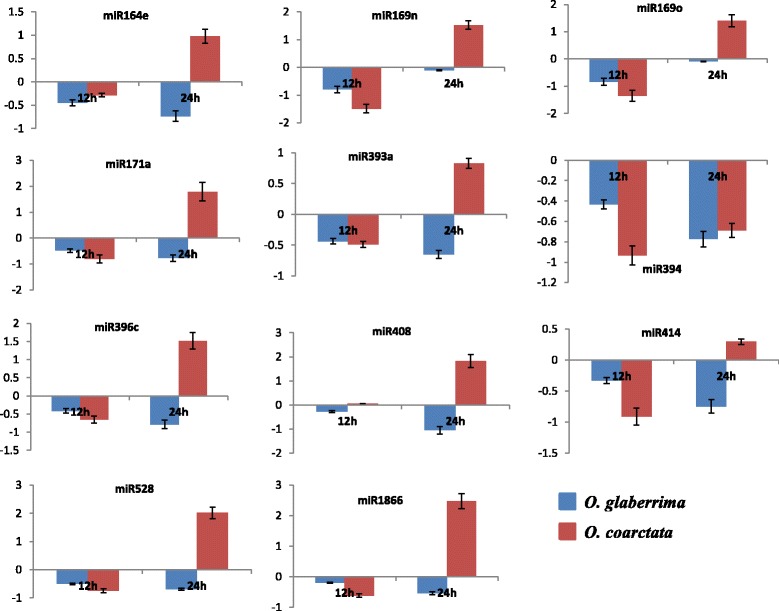
qPCR based expression profiles. Histograms showing the mean fold change (log_2_ scale) in the expression of 11 salt-responsive miRNAs at vegetative stage of *O. coarctata* and *O. glaberrima* under 12 h and 24 h of salinity stress. Expression of all the studied miRNAs was downregulated both at 12 h and 24 h of salinity stress in *O. glaberrima,* whereas the expression of all the miRNAs, except miR394, can be seen upregulated only at 24 h of salt stress in *O. coarctata*. Error bars represent the standard deviation (±), *n* = 3

To know if the different sequence variants or alleles of some known salt-responsive miRNA genes, identified in the different *Oryza* species through our *in-silico* analysis, have any impact on their expression, we analysed the qPCR-based expression patterns of these miRNAs at vegetative stage in the two *Oryza* species namely *O. coarctata* and *O. glaberrima* under salinity stress. The expression of the studied miRNAs in rice has already been known under salinity (Additional file 18). Additionally, the miRNA genes of *O. coarctata* have been identified in our previous study [67]. While the results showed that the overall expression of all the studied miRNAs was downregulated both at 12 and 24 h of salinity treatment in *O. glaberrima,* the downregulation was more at 24 h than 12 h for almost all miRNAs, indicating that down regulation of such miRNAs in this species is important in probably protecting it from the salinity stress. In contrast, the expression of all miRNAs in *O. coarctata* was downregulated at 12 h of stress, whereas their expression (except miR394) was significantly upregulated (*p >* 0.05) when the timing of stress was increased from 12 to 24 h. Only miR394 was found to be downregulated under salinity stress in both the species studied, implying that this miRNA might be the negative regulator of salinity stress which is supported by the results of an earlier study [68]. The expression of most of the studied salt-responsive miRNAs in *O. coarctata* at 24 h indicated that they may not be induced immediately after the imposition of stress. The upregulation of these miRNAs in the halophytic rice relative under salinity reflects the fact that extensive reprogramming in the transcriptional machinery under salinity confers the tolerance to salinity in *O. coarctata*. The differential behaviour in the expression of a same miRNAs in two *Oryza* species harmonizes with some earlier reports [9, 69]. The reason for the different expression patterns of the studied salt-responsive miRNA orthologues in *O. glaberrima*, *O. coarctata* and rice could not be known. However, it might be attributed to the different sequence variants or alleles of the corresponding miRNA loci in the two *Oryza* species and rice. The observation of different expression patterns of miRNAs in the two species is also justified by the results of a previous report which states that the transcripts of *O. coarctata* and rice exhibit a low similarity with each other probably due to their evolutionary divergence, suggesting considerable differences in their expression too [70]). The relative transcript abundance of the studied miRNAs under salinity has been presented as bar graphs (Fig. 7).

## Conclusions

In the present study, we identified the orthologous miRNA genes of rice from the ten different *Oryza* species and performed a comprehensive evolutionary analysis in terms of rate of sequence variations and selection pressures on these orthologous genes. The species-specific gain and loss of miRNA genes and their duplicated paralogues signified the importance of gene duplication in the birth of new genes which might have led to phenotypic divergence and hence in the evolution of the *Oryza* genomes. The extensive rearrangements in the genomic order, differential amplification patterns of transposable elements, variations in the precursor sequences than the variations in purifyingly selected mature and star sequences as well as differential frequency of transition substitutions in precursors of miRNA genes may act as crucial contributory factors in the divergence and evolution of *Oryza*. Hence, diverged forces might link genomic rearrangements, sequence variants and base content with the conservation in miRNA genes of *Oryza*. The non-neutral selection at numerous miRNA loci suggested a recent selective sweep and/or purifying selection in the *Oryza* genomes. The loss of average sequence diversity in domestic rice during the course of domestication at different miRNA loci strongly alludes that the wild species of rice are potential genetic resources that are significantly untapped from the perspective of crop improvement. These sequence variants might eventually lead to the difference in the expression patterns under specific developmental and stress conditions which is evidenced by our expression study in *O. glaberrima* and *O. coarctata*. It hence attests that nature has focussed significantly on the miRNAs as the one of the main elements for the differential accumulation of variations in populations under selection pressures.

## Methods

### Sequence identification from different Oryza species

In order to identify the homologous sequences of rice (*O. sativa* subsp. *japonica*) in the 10 different *Oryza* species, we first downloaded all the miRNA precursor sequences of rice from the miRBase (Release 21.0, June 2014). These sequences were used as queries for performing the BLASTn search [71] against each of the 10 *Oryza* species (*E*-value 10^−4^) in gramene [72]. Criteria described by some previous studies [38, 73–75] were used for assigning a BLAST output hit as a potential precursor. These are i) BLAST hit should not be more than 20 nt lesser than the query and should have an *E*-value of ≤10^−4^, ii) the mismatches in the mature sequence of desired sized hit with the mature sequence of rice should be ≤2, iii) the sequence should adopt an appropriate hairpin structure when analysed by RNA Fold [76], iv) the minimum free energy of the predicted hairpin structure should be ≤ −15 kcal/mol, v) the maximum bulge size in the hairpin should not be more than 12, vi) the maximum number of mismatches allowed between miRNA and miRNA^*^ is 6.

The sequences of all miRNA precursors from the 10 *Oryza* species and their other details are shown in Additional file 1.

### Plant materials and PCR reactions for sequencing of miR1861 clusters

miR1861 gene family is one of the miRNA gene families that might be organized into distinct compact clusters, and can be transcribed as single units. The idea of existence of this gene family into clusters was appreciated due to most of its members occurring as tandem and polycistronic miRNAs [35, 64]. Therefore, we attempted to isolate the clusters of this family from the four *Oryza* species (*O. sativa* sp. *indica*, *O. glaberrima, O. nivara* and *O. rufipogon*) with the *O. brachyantha* as the negative control as none of the genes from this family was found in *O. brachyantha* by our *in-silico* results (Additional file 1). Genomic DNA was extracted from the leaf samples of these *Oryza* species as described in our earlier study [9], and was used as a template for amplification of miR1861 clusters from each genotype using cluster-specific primers (Additional file 19) in C1000 Touch™ thermal cycler (Bio-Rad Laboratories, Hercules, CA, USA). The parameters for the PCR reaction were: 5 min at 95 °C, 45 cycles of 15 s at 95 °C, 30 s at varying annealing temperatures and 30 s at 72 °C. The amplification products were cloned in pGEMT-easy vector, transformed into DH5α and sequenced. For each cluster, primers were designed from the flanking regions of individual pre-miRNA members (e.g. in 2-membered cluster-II, the primers were designed from upstream of ‘b’ and downstream of ‘c’ so that both members ‘b’ and ‘c’ including the genomic region between them was amplified). Precursor sequences of individual members of osa-miR1861 clusters were determined from the miRBase, and then each cluster sequence was used as a query to extract miR1861 clusters from the other *Oryza* species.

### Multiple sequence alignment and phylogenetic analysis

In order to analyse the sequence convergence or divergence in nine sequenced miR1861 clusters from the five *Oryza* species, each cluster from all these species was aligned using Clustal X 1.83 with default parameters. The phylogenetic tree was constructed using Clustal Omega [77], whereas dendrograms from a conserved and a non-conserved miRNA were constructed using MEGA 6 [78] with the Neighbor-joining method [79].

### Stress treatments and quantitative PCR-based expression analysis of miRNA sequence variants

In order to find out the relation between the sequence variations of some miRNA loci and their corresponding probable expression variations, we selected 11 known salt responsive miRNAs based on the published literature [80, 81] for studying their expression in two wild species of rice namely *O. coarctata* and *O. glaberrima* under salinity stress. While *O. coarctata* is a halophytic wild relative of rice that grows normally under highly saline conditions [67], *O. glaberrima* is a well-adapted and cultivable species of *Oryza* in West Africa that has acquired traits for increased biotic and abiotic stress tolerance [82]. The young plants (at tillering stage) of *O. coarctata* and *O. glaberrima* were given a salt stress by keeping them submerged in 450 mM and 200 mM NaCl solutions respectively, for 24 h. Plants treated with distilled water for 24 h were considered as control. After 24 h, the leaf tissues were harvested from both salt stressed and control plants in three biological replicates and frozen until further use. The isolation of miRNA, cDNA synthesis and qPCR analysis for all the salt-responsive miRNAs was performed as per our previous study [9]. The details of primers are given in Additional file 19.

### Evaluation of substitution rates of miRNA genes from Oryza species

In order to estimate the substitution rates in the miRNA genes of 10 *Oryza* species, the sequences of mature, star (miRNA-complementary region) and precursor regions of 32 conserved and 22 non-conserved miRNA genes representing 19 families each were taken [64, 83, 84]. They were aligned separately using clustalW in MEGA 6. For miRNA members having more than one hit or paralogue in a species, all the hits were taken for the analysis. Initially, the average numbers of nucleotide substitutions per site *N* for each region were calculated [85] among the particular miRNA genes from the 10 *Oryza* species. The *N* was then used to estimate the substitution rate (R) for a particular region of miRNA gene using the formula R = *N*/2 T, where T is the divergence time of *Oryza* which is 15 MY [28]. In order to see whether the rate of nucleotide substitutions in the precursor sequences of conserved and non-conserved miRNA genes was biased more towards transitions or transversions, the transition/transversion ratios [86] were calculated for each precursor from the all *Oryza* species. To know the probability of substitution from one base to another base, patterns of all transition and transversion substitutions were estimated [87]. Further, the conservation in the different regions (left, middle and right regions) of mature and corresponding star sequences of the above miRNA genes was analyzed.

To compare with the studied miRNA genes, the substitution rates at synonymous (dS) and non-synonymous (dN) sites in all the orthologous protein coding genes of 10 *Oryza* species were determined using GrameneMart [88]. The average dN/dS ratios were calculated across the 10 *Oryza* species which were then used for computing the substitution rate in the same manner as done for miRNA genes.

### Tests of neutrality

The neutrality of miRNA sequence polymorphisms among *Oryza* was assessed by means of neutrality tests such as, Tajima’s *D* [57] and Fu and Li’s *F* [58] performed on 80 different miRNA loci using the software DnaSP v5.10 [89]. Based on the segregating sites in the pre-miRNA sequences of *Oryza*, the values for the two neutrality tests were calculated.

## Additional files

Additional file 1: Details of different orthologous miRNAs of *O. sativa* (*japonica*) from the ten diploid *Oryza* species. oba- (*O. barthi*), obr- (*O. brachyantha*), ogl- (*O. glaberrima*), oglu- (*O. glumaepatula*), olo- (*O.longistaminata*), ome- (*O. meridionalis*), oni- (*O. nivara*), opu- (*O. punctata*), oru- (*O. rufipogon*), osi- (*O. sativa* subsp. *indica*). (XLSX 867 kb)

Additional file 2: Stemloop structures of conserved-miR394 (A) and non-conserved-miR1866 (B) from 10 *Oryza* species. (PPTX 1309 kb)

Additional file 3: No. of intragenic and intergenic miRNA genes with and without paralogous duplicates in the *Oryza* species. (XLSX 10 kb)

Additional file 4: Average no. of nucleotide substitutions per site in different regions of miRNA genes, transition/transversion bias as well as rates of nucleotide substitutions in conserved and non-conserved miRNA genes along with all the protein coding genes of *Oryza*. JC-values (Jukes Cantor-values). (XLSX 12 kb)

Additional file 5: Sequence conservation in the precursors of non-conserved miR528 (A) and conserved miR393a (B) among the *Oryza* species. It can be seen from the alignments that sequence regions around the mature sequence are more conserved than other regions in the precursors of both miRNAs. Multiple sequence alignment of FLZ proteins of selected species is generated using CLUSTAL X and visualized in Jalview. (PPTX 85 kb)

Additional file 6: *Ka/Ks* ratios of all orthologous protein-coding genes of 10 *Oryza* species. Ratios were calculated using GrameneMart. (XLSX 9 kb)

Additional file 7: Transition/transversion ratio. Nucleotide substitutions in both conserved and non-conserved miRNAs favor transitions over transversions. Higher ratio can be seen in case of non-conserved miRNAs compared to conserved ones. (PPTX 51 kb)

Additional file 8: Different types of transition and transversion nucleotide substitutions in the precursor sequences of the selected conserved and non-conserved miRNA genes from all the studied *Oryza* species. (XLSX 13 kb)

Additional file 9: Proportion of conserved sites in the different parts of mature and star regions of the studied conserved and non-conserved miRNAs from the 10 *Oryza* species. If nucleotides in a certain part of the mature or star sequences were completely conserved among *Oryza*, it was given a value of 1. (XLSX 11 kb)

Additional file 10: Relation between the base content of miRNA genes and their conservation. AT-content can be seen as lesser than GC-content in mature and star regions of both conserved as well as non-conserved miRNA genes. It also depicts that AT-content in non-conserved miRNA precursors is higher than the conserved ones. (PPTX 62 kb)

Additional file 11: Average of base composition (AT- and GC-content) of mature, star and precursor regions of the selected conserved and non-conserved miRNA genes of *Oryza*. (XLSX 13 kb)

Additional file 12: The values of Tajima’s *D* and Fu and Li’s *F* as well as nucleotide diversity for each of the 80 miRNA loci (pre-miRNAs) from the 10 *Oryza* species. The parameters were computed using software DnaSP v5.10. (XLSX 12 kb)

Additional file 13: The average nucleotide sequence diversity (*π*) at the 11 miRNA loci (showing significantly negative values for both the neutrality tests) in cultivated rice and wild *Oryza* species. The figure depicts that nucleotide diversity at these significant loci has been considerably lost during the domestication. (PPTX 51 kb)

Additional file 14: The average sequence diversity (*π*) at the 11 miRNA loci (with the significantly negative values for *D* and *F*) in cultivated rice and wild species of *Oryza*. (XLSX 9 kb)

Additional file 15: Multiple sequence alignment of all the sequencedmiR1861-clusters individually shows the significant conservation in the cluster sequences among the *Oryza* species. The presence of distinct nucleotide substitutions and indels were also evident with the Cluster-VII being the most diverged. (TXT 20 kb)

Additional file 16: Structure of the 2-membered clusters of miR1861 family. Here cluster-II represents all the 2-membered miR1861-clusters. The two members of a 2-membered cluster are joined by a nt. sequence of ~100 bp (A). Alignment of cluster-II from the *Oryza* species (B) showing the deletion of 22 nt is consistently present in the 2nd member of a 2-membered cluster. (PPTX 343 kb)

Additional file 17: Percent identity matrix showing the identity among the nine different clusters of osa-miR1861 family in rice. (TXT 1003 bytes)

Additional file 18: Regulation of the expression of 11 salt-responsive miRNAs of rice under salinity stress. (DOCX 15 kb)

Additional file 19: Details of different types of primer sequences used in the present study. (DOCX 17 kb)
