# Supplementary material for: Comprehensive survey and evolutionary analysis of genome-wide miRNA genes from ten diploid Oryza species
Source: BMC Genomics. 2017 Sep 11;18:711. doi: 10.1186/s12864-017-4089-4 (PMC5594537; doi:10.1186/s12864-017-4089-4)
Supplement: Supplementary file 2 — Stemloop structures of conserved-miR394 (A) and non-conserved-miR1866 (B) from 10 Oryza species. (PPTX 1309 kb) [file 12864_2017_4089_MOESM2_ESM.pptx]

## Slide 1
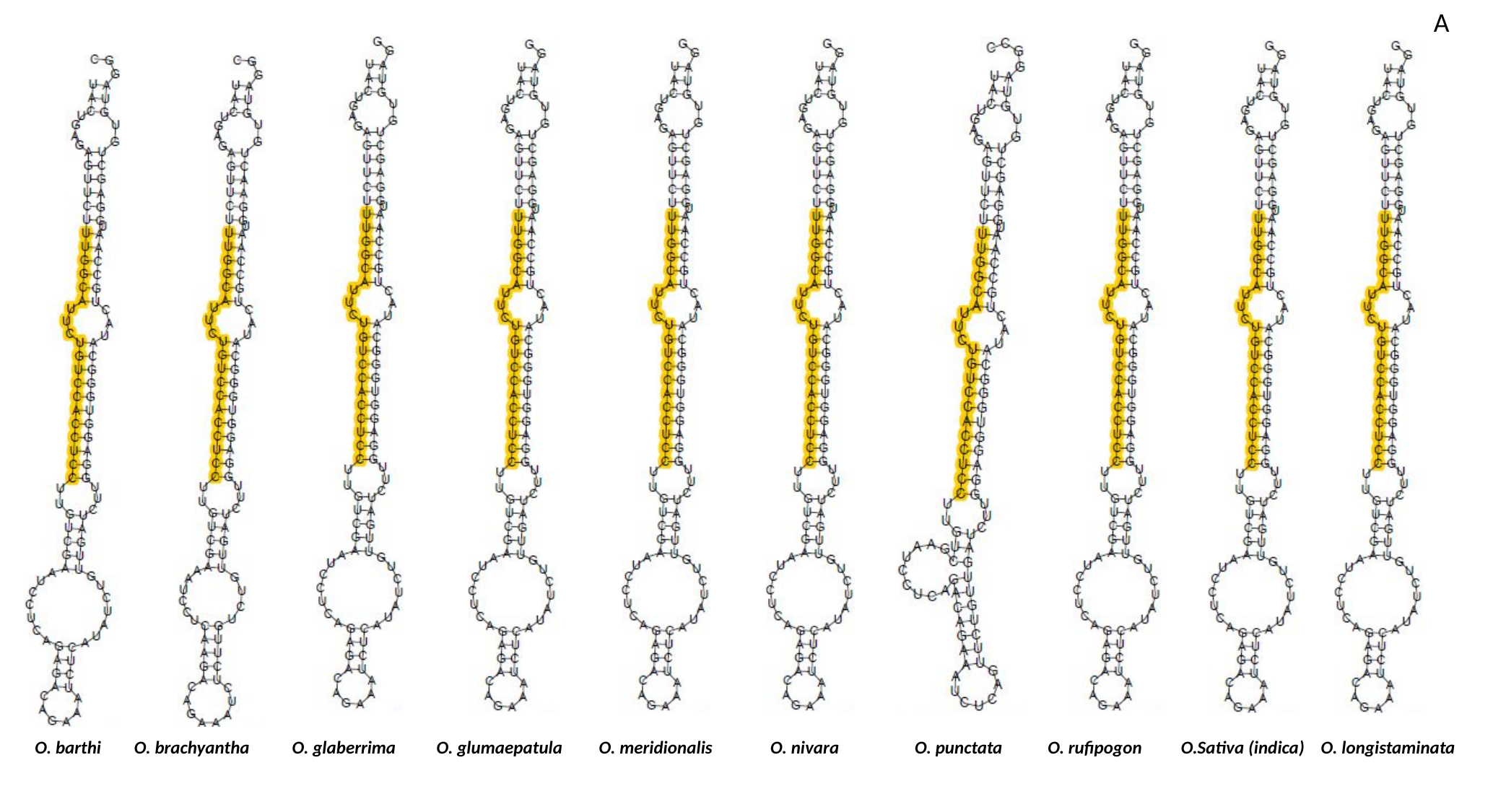

A
O. barthi
O. brachyantha
O. glaberrima
O. glumaepatula
O. meridionalis
O. nivara
O. punctata
O. rufipogon
O.Sativa (indica)
O. longistaminata

## Slide 2
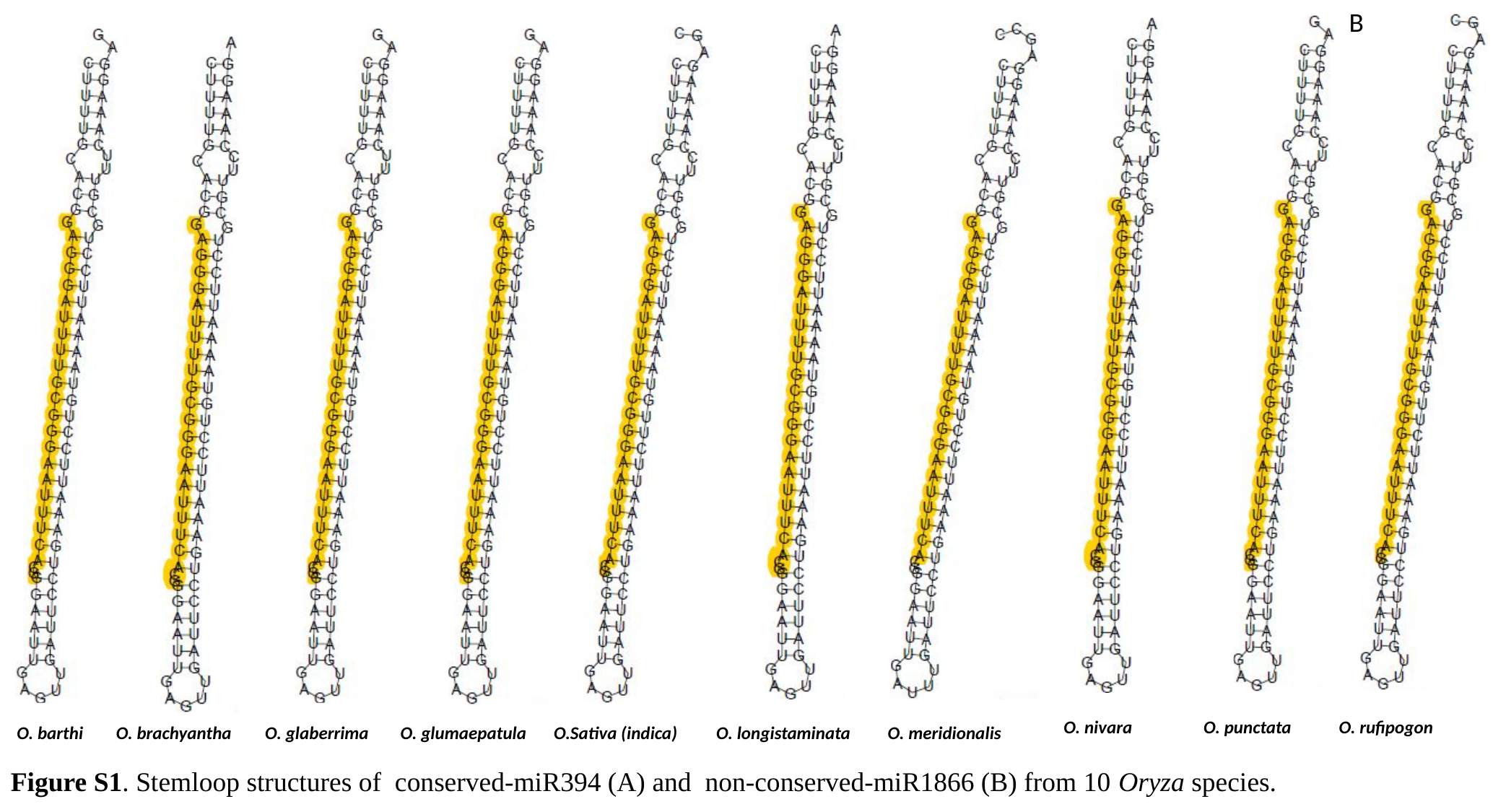

B
O. nivara
O. punctata
O. rufipogon
O. barthi
O. brachyantha
O. glaberrima
O. glumaepatula
O.Sativa (indica)
O. longistaminata
O. meridionalis
Figure S1. Stemloop structures of conserved-miR394 (A) and non-conserved-miR1866 (B) from 10 Oryza species.
