# Supplementary material for: Comprehensive survey and evolutionary analysis of genome-wide miRNA genes from ten diploid Oryza species
Source: BMC Genomics. 2017 Sep 11;18:711. doi: 10.1186/s12864-017-4089-4 (PMC5594537; doi:10.1186/s12864-017-4089-4)
Supplement: Supplementary file 5 — Sequence conservation in the precursors of non-conserved miR528 (A) and conserved miR393a (B) among the Oryza species. It can be seen from the alignments that sequence regions around the mature sequence are more conserved than other regions in the precursors of both miRNAs. Multiple sequence alignment of FLZ proteins of selected species is generated using CLUSTAL X and visualized in Jalview. (PPTX 85 kb) [file 12864_2017_4089_MOESM5_ESM.pptx]

## Slide 1
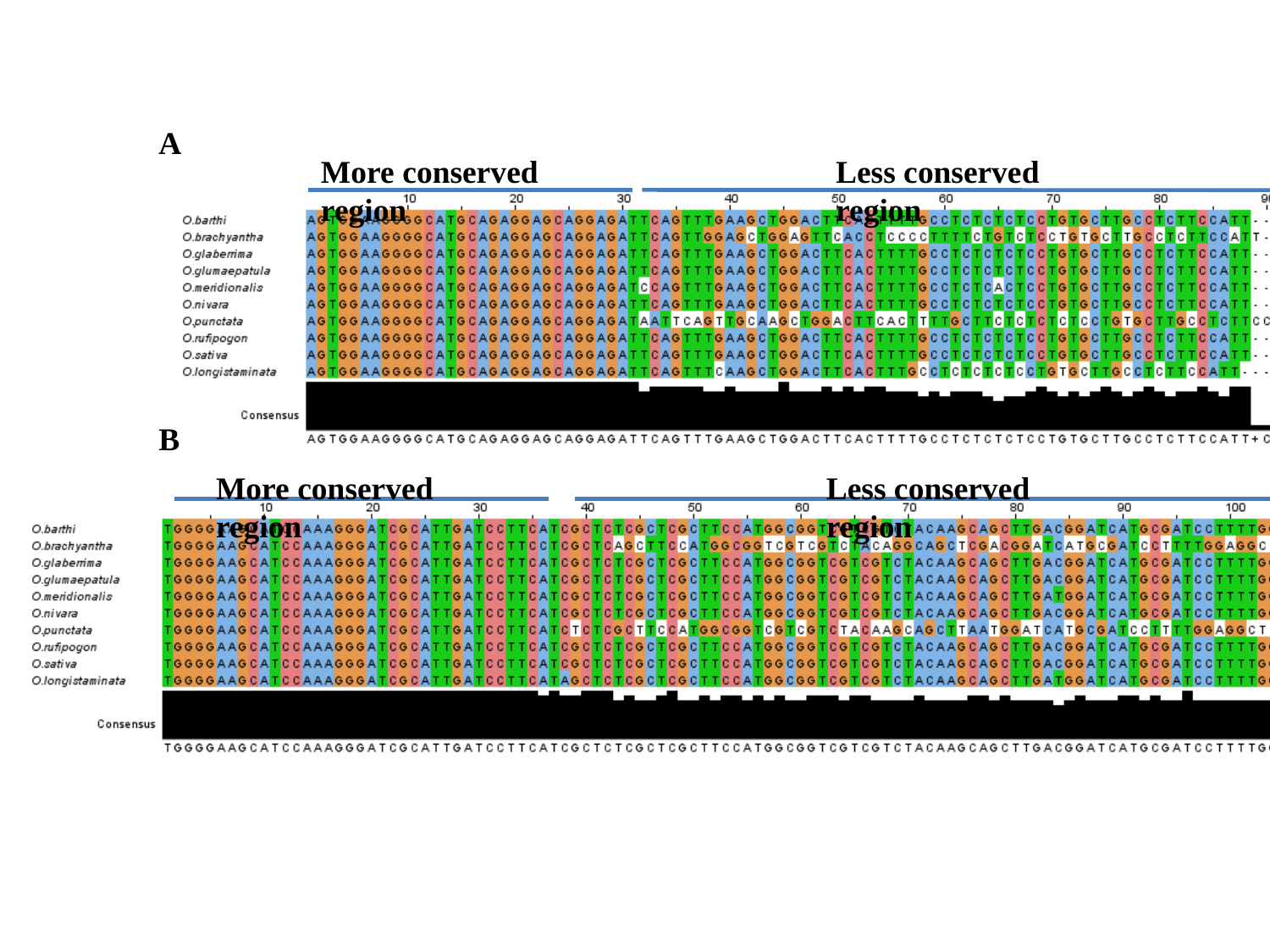

A
More conserved region
Less conserved region
More conserved region
Less conserved region
B

## Slide 2
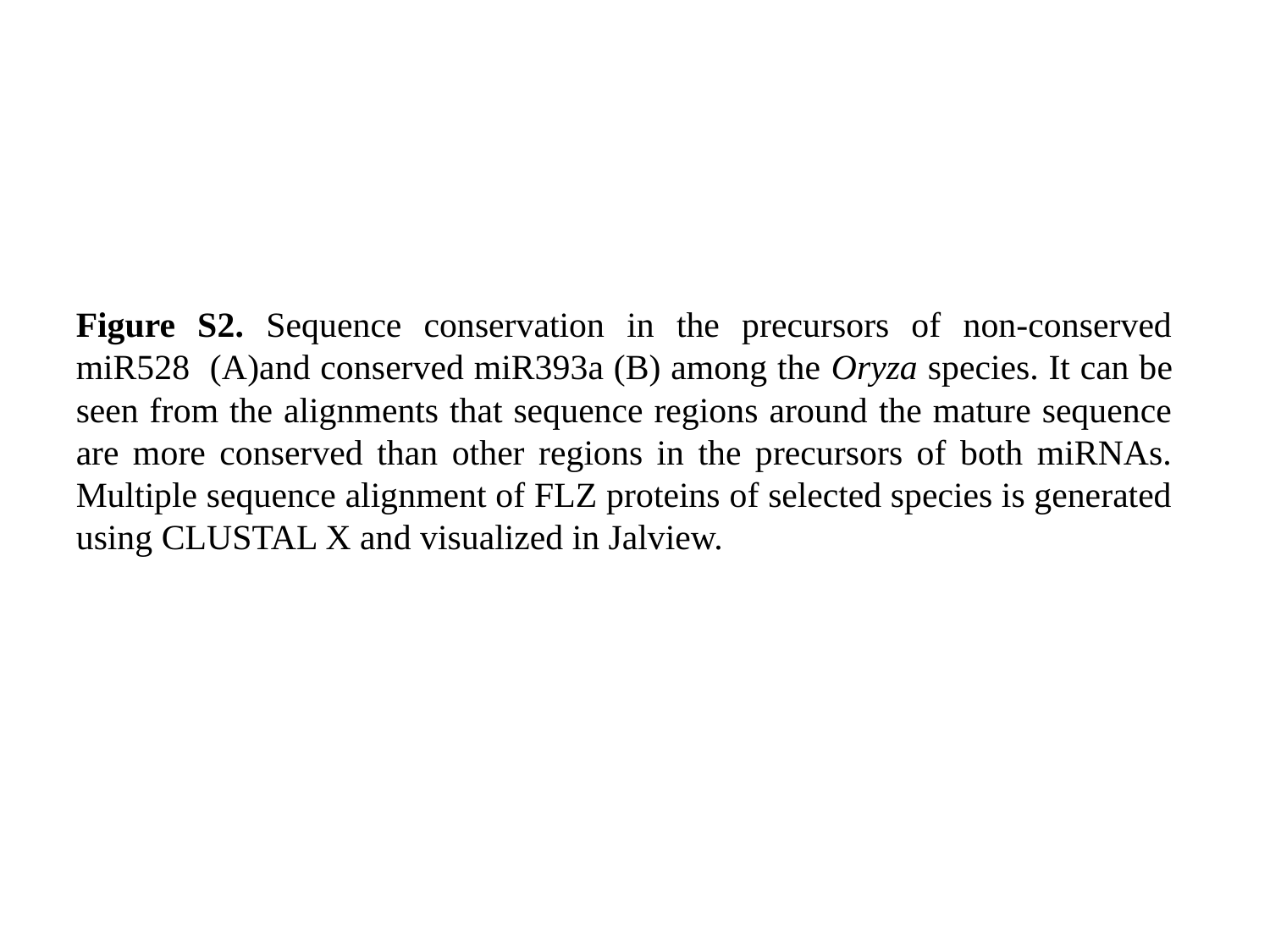

Figure S2. Sequence conservation in the precursors of non-conserved miR528 (A)and conserved miR393a (B) among the Oryza species. It can be seen from the alignments that sequence regions around the mature sequence are more conserved than other regions in the precursors of both miRNAs. Multiple sequence alignment of FLZ proteins of selected species is generated using CLUSTAL X and visualized in Jalview.
