# Supplementary material for: Comprehensive survey and evolutionary analysis of genome-wide miRNA genes from ten diploid Oryza species
Source: BMC Genomics. 2017 Sep 11;18:711. doi: 10.1186/s12864-017-4089-4 (PMC5594537; doi:10.1186/s12864-017-4089-4)
Supplement: Supplementary file 7 — Transition/transversion ratio. Nucleotide substitutions in both conserved and non-conserved miRNAs favor transitions over transversions. Higher ratio can be seen in case of non-conserved miRNAs compared to conserved ones. (PPTX 51 kb) [file 12864_2017_4089_MOESM7_ESM.pptx]

## Slide 1
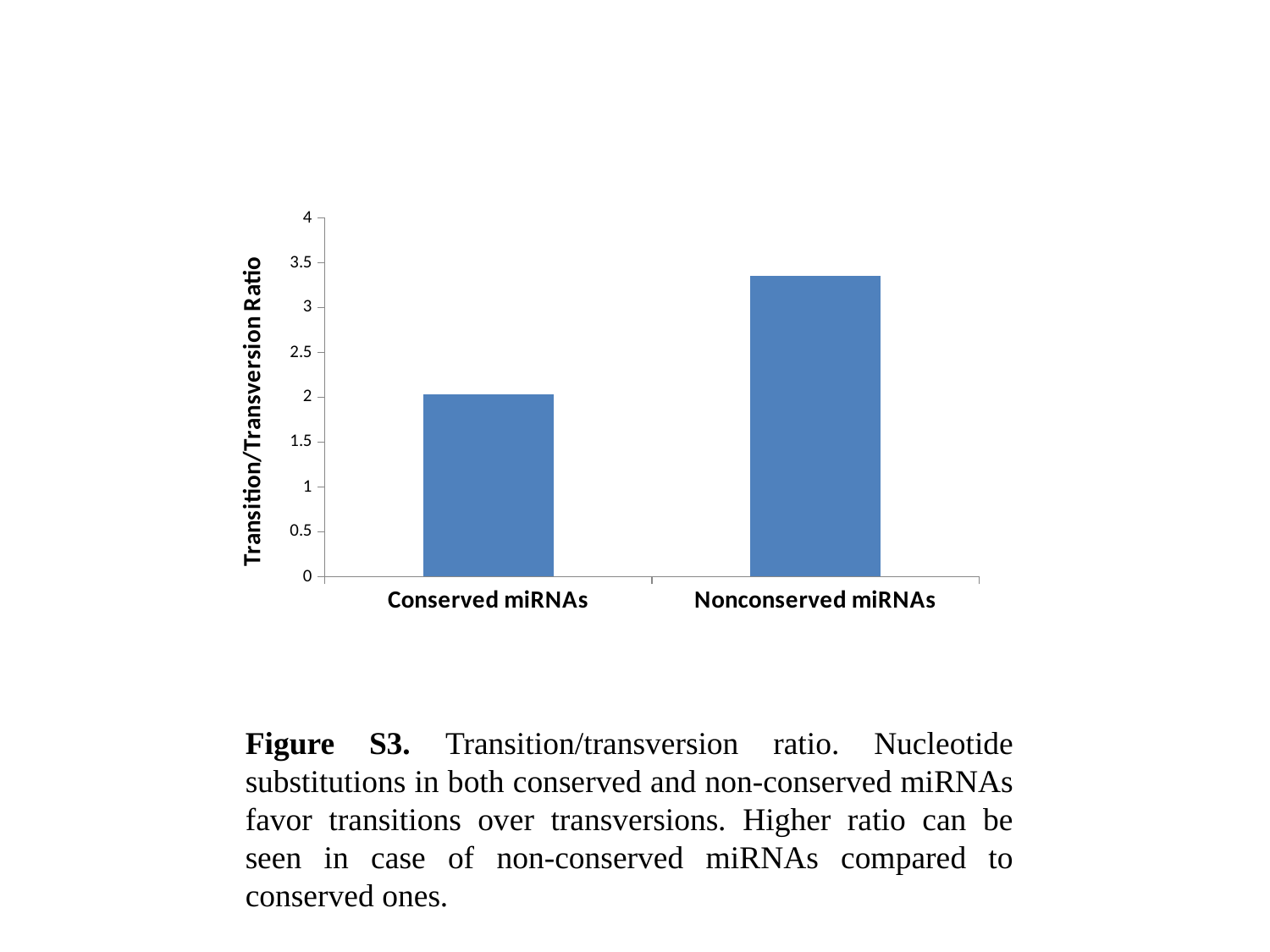

### Chart
| Category | |
|---|---|
| Conserved miRNAs | 2.0334375 |
| Nonconserved miRNAs | 3.357142857142857 |Figure S3. Transition/transversion ratio. Nucleotide substitutions in both conserved and non-conserved miRNAs favor transitions over transversions. Higher ratio can be seen in case of non-conserved miRNAs compared to conserved ones.
