# Supplementary material for: Comprehensive survey and evolutionary analysis of genome-wide miRNA genes from ten diploid Oryza species
Source: BMC Genomics. 2017 Sep 11;18:711. doi: 10.1186/s12864-017-4089-4 (PMC5594537; doi:10.1186/s12864-017-4089-4)
Supplement: Supplementary file 10 — Relation between the base content of miRNA genes and their conservation. AT-content can be seen as lesser than GC-content in mature and star regions of both conserved as well as non-conserved miRNA genes. It also depicts that AT-content in non-conserved miRNA precursors is higher than the conserved ones. (PPTX 62 kb) [file 12864_2017_4089_MOESM10_ESM.pptx]

## Slide 1
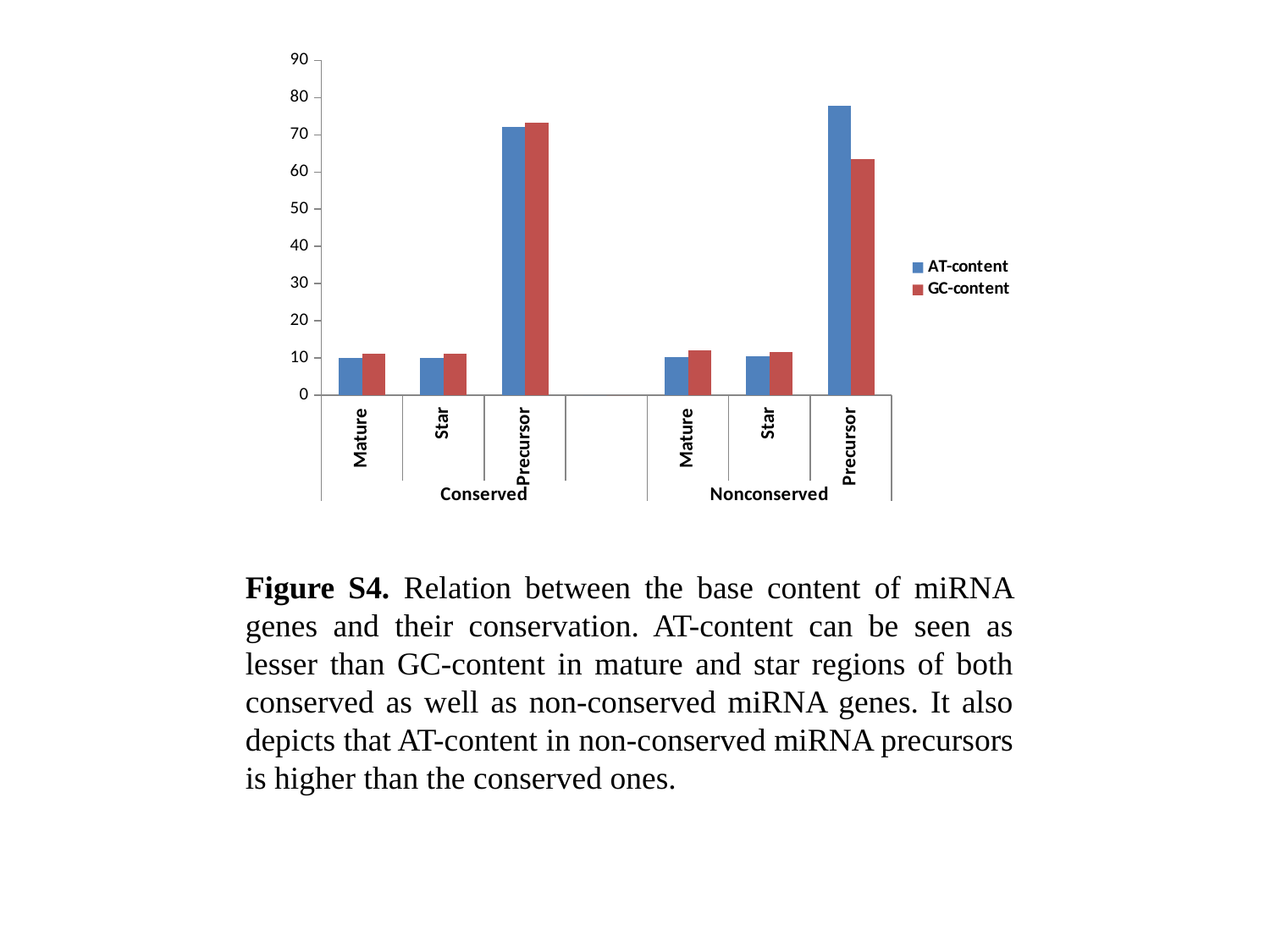

### Chart
| Category | AT-content | GC-content |
|---|---|---|
| Mature | 10.02222 | 11.105793450881615 |
| Star | 10.035805626598465 | 11.12531969309463 |
| Precursor | 72.1479099678457 | 73.17684887459798 |
| | 0.0 | 0.0 |
| Mature | 10.151785714285717 | 12.053571428571425 |
| Star | 10.366071428571425 | 11.61160714285714 |
| Precursor | 77.70089285714283 | 63.41964285714284 |Figure S4. Relation between the base content of miRNA genes and their conservation. AT-content can be seen as lesser than GC-content in mature and star regions of both conserved as well as non-conserved miRNA genes. It also depicts that AT-content in non-conserved miRNA precursors is higher than the conserved ones.
