# Supplementary material for: Comprehensive survey and evolutionary analysis of genome-wide miRNA genes from ten diploid Oryza species
Source: BMC Genomics. 2017 Sep 11;18:711. doi: 10.1186/s12864-017-4089-4 (PMC5594537; doi:10.1186/s12864-017-4089-4)
Supplement: Supplementary file 13 — The average nucleotide sequence diversity (π) at the 11 miRNA loci (showing significantly negative values for both the neutrality tests) in cultivated rice and wild Oryza species. The figure depicts that nucleotide diversity at these significant loci has been considerably lost during the domestication. (PPTX 51 kb) [file 12864_2017_4089_MOESM13_ESM.pptx]

## Slide 1
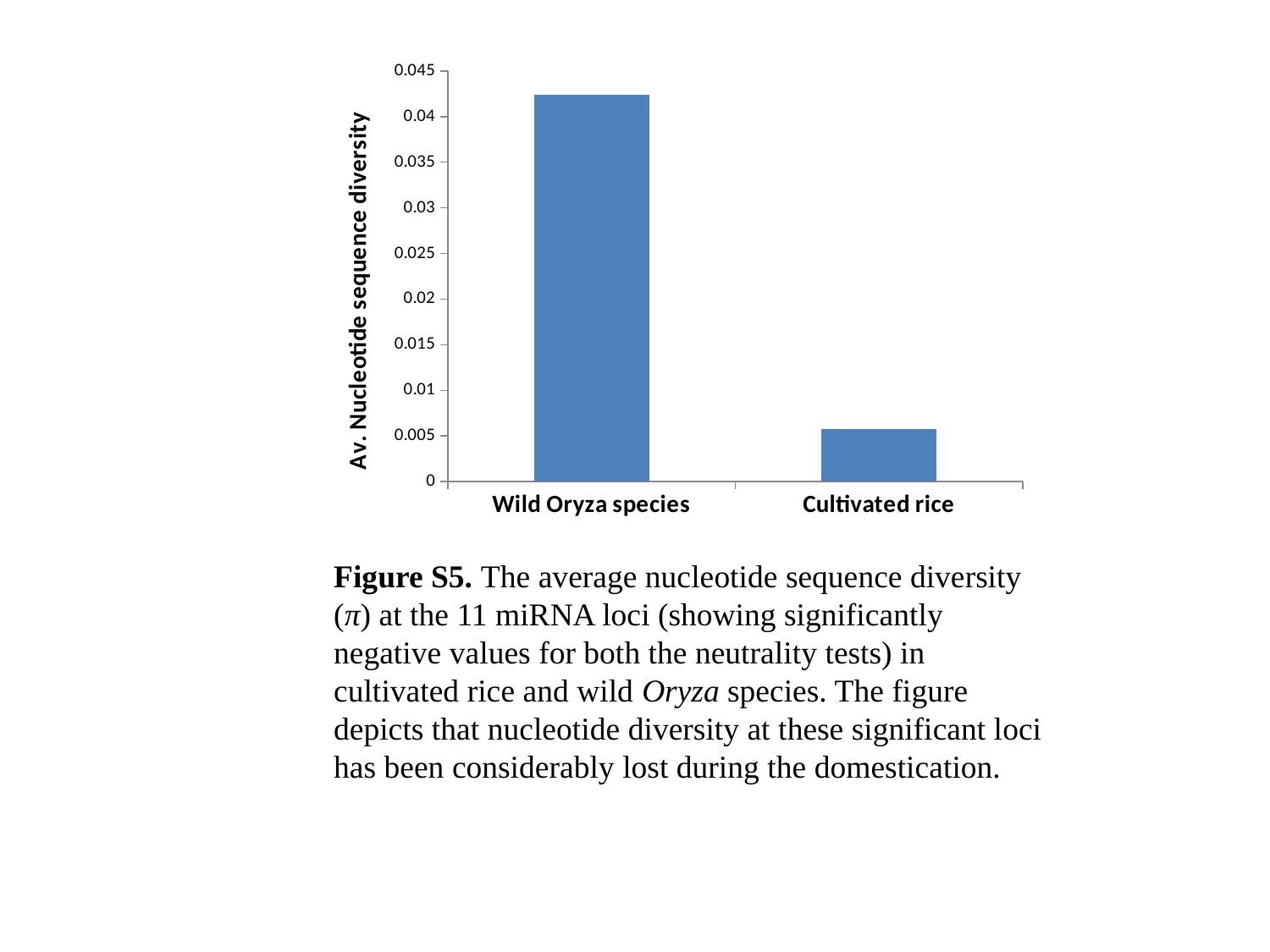

### Chart
| Category | |
|---|---|
| Wild Oryza species | 0.04239454545454548 |
| Cultivated rice | 0.005751818181818184 |Figure S5. The average nucleotide sequence diversity (π) at the 11 miRNA loci (showing significantly negative values for both the neutrality tests) in cultivated rice and wild Oryza species. The figure depicts that nucleotide diversity at these significant loci has been considerably lost during the domestication.
