# Supplementary material for: Comprehensive survey and evolutionary analysis of genome-wide miRNA genes from ten diploid Oryza species
Source: BMC Genomics. 2017 Sep 11;18:711. doi: 10.1186/s12864-017-4089-4 (PMC5594537; doi:10.1186/s12864-017-4089-4)
Supplement: Supplementary file 16 — Structure of the 2-membered clusters of miR1861 family. Here cluster-II represents all the 2-membered miR1861-clusters. The two members of a 2-membered cluster are joined by a nt. sequence of ~100 bp (A). Alignment of cluster-II from the Oryza species (B) showing the deletion of 22 nt is consistently present in the 2nd member of a 2-membered cluster. (PPTX 343 kb) [file 12864_2017_4089_MOESM16_ESM.pptx]

## Slide 1
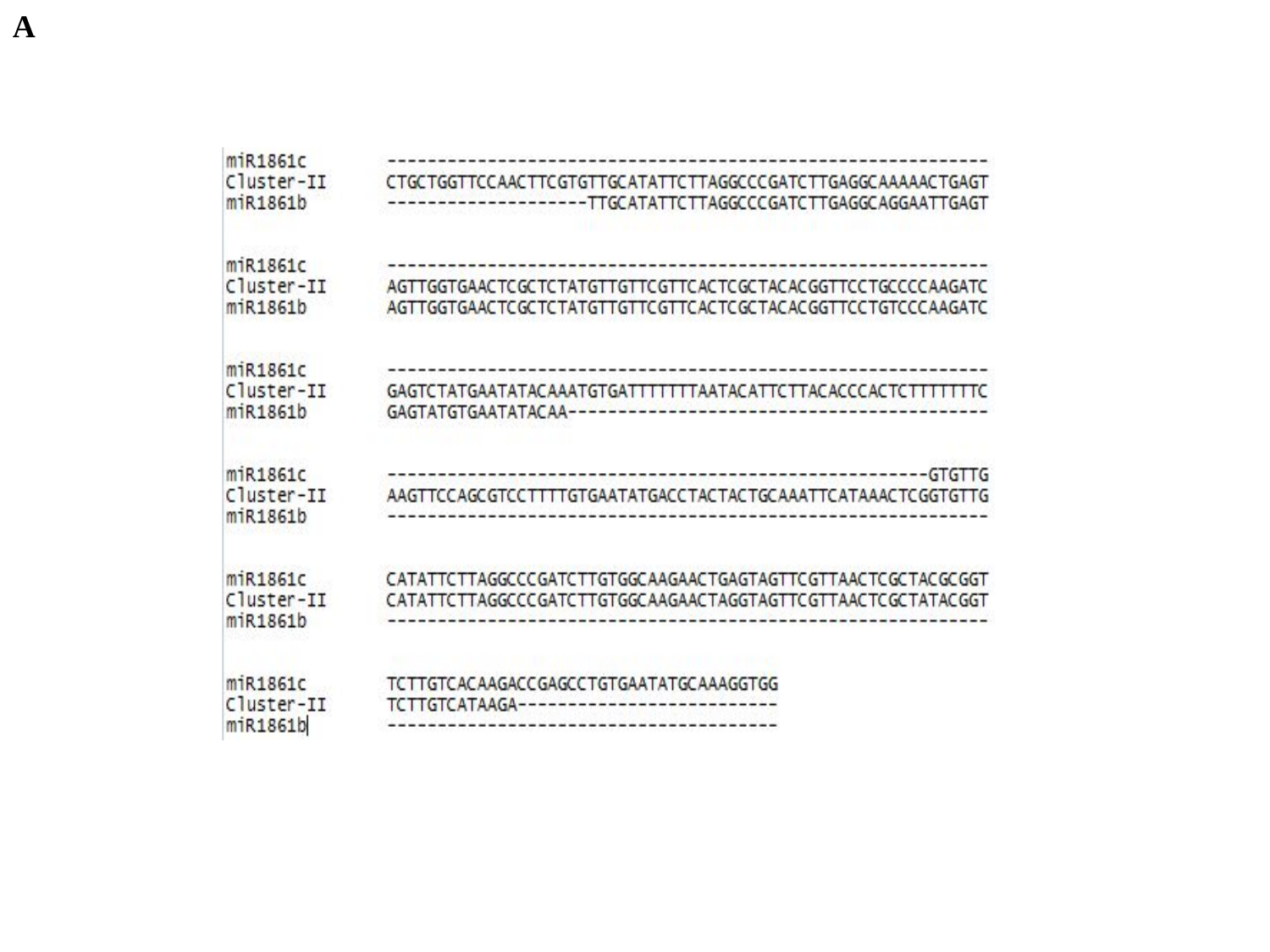

A

## Slide 2
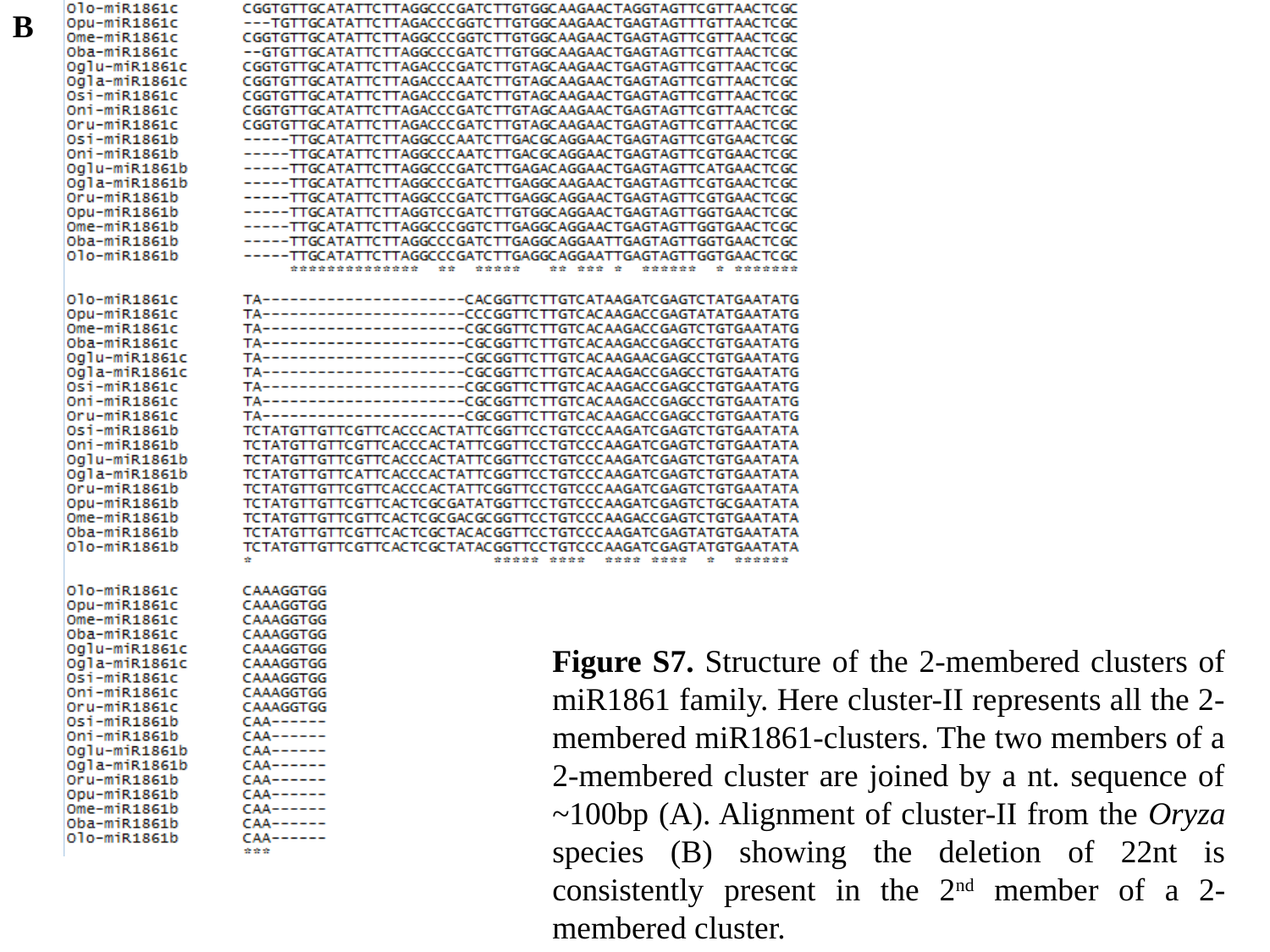

B
Figure S7. Structure of the 2-membered clusters of miR1861 family. Here cluster-II represents all the 2-membered miR1861-clusters. The two members of a 2-membered cluster are joined by a nt. sequence of ~100bp (A). Alignment of cluster-II from the Oryza species (B) showing the deletion of 22nt is consistently present in the 2nd member of a 2-membered cluster.
