# Supplementary material for: Comprehensive survey and evolutionary analysis of genome-wide miRNA genes from ten diploid Oryza species
Source: BMC Genomics. 2017 Sep 11;18:711. doi: 10.1186/s12864-017-4089-4 (PMC5594537; doi:10.1186/s12864-017-4089-4)
Supplement: Supplementary file 18 — Regulation of the expression of 11 salt-responsive miRNAs of rice under salinity stress. (DOCX 15 kb) [file 12864_2017_4089_MOESM18_ESM.docx]

| **miRNA** | **Regulation** | **Reference** |
| --- | --- | --- |
| osa-miR528 | Upregulated | Sunkar et al. (2008), Yuan et al. (2015) |
| osa-miR169n | Upregulated | Zhao et al. (2009) |
| osa-miR393a | Downregulated | Ganie et al. (2016) |
| osa-miR394 | Downregulated | Song et al. (2013) |
| osa-miR396c | Downregulated | Gao et al. (2010) |
| osa-miR414 | Downregulated | Macovei and Tuteja (2012) |
| osa-miR408 | Downregulated | Macovei and Tuteja (2012) |
| osa-miR164e | Downregulated | Macovei and Tuteja (2012) |
| osa-miR171a | Downregulated | Sunkar et al. (2008) |
| osa-miR1866 | Downregulated | Barrera-Figueroa et al. (2012) |

Table S12. Regulation of the expression of 11 salt-responsive miRNAs of rice under salinity stress.

Barrera-Figueroa BE, et al. 2012, High throughput sequencing reveals novel and abiotic stress-regulated microRNAs in the inflorescences of rice. BMC Plant Biol. 12:1.

Ganie SA, Dey N, Mondal TK. 2016, Promoter methylation regulates the abundance of osa-miR393a in contrasting rice genotypes under salinity stress. Funct. Integr. Genomics 16: 1-11.

Gao, P, et al. 2010, Over-expression of *osa-MIR396c* decreases salt and alkali stress tolerance. Planta 231: 991-1001.

Macovei A, Tuteja N. 2012, microRNAs targeting DEAD-box helicases are involved in salinity stress response in rice (*Oryza sativa* L.). BMC Plant Biol. 12: 183-195.

Song JB, et al. 2013, miR394 and LCR are involved in *Arabidopsis* salt and drought stress responses in an abscisic acid-dependent manner. BMC Plant Biol. 13: 210.

Sunkar, R, et al. 2008, Identification of novel and candidate miRNAs in rice by high throughput sequencing. BMC Plant Biol. 8: 1.

Yuan S, et al. 2015, Constitutive expression of rice *MicroRNA528* alters plant development and enhances tolerance to salinity stress and nitrogen starvation in Creeping Bentgrass. Plant Physiol*.* 169: 576-593.

Zhao B, et al. 2009, Members of miR-169 family are induced by high salinity and transiently inhibit the NF-YA transcription factor. BMC Mol. Biol. 10: 29-39.
