# Supplementary material for: Comprehensive survey and evolutionary analysis of genome-wide miRNA genes from ten diploid Oryza species
Source: BMC Genomics. 2017 Sep 11;18:711. doi: 10.1186/s12864-017-4089-4 (PMC5594537; doi:10.1186/s12864-017-4089-4)
Supplement: Supplementary file 19 — Details of different types of primer sequences used in the present study. (DOCX 17 kb) [file 12864_2017_4089_MOESM19_ESM.docx]

| Primer | Forward (5´-3´) | Reverse (5´-3´) | T_m_(^o^C) |
| --- | --- | --- | --- |
| **miR1861-cluster specific primers** | | | |
| CLUSTER-I (miR1861a) | CAACCAAACCTAGCACACAACTAC | TACAAACGGATGGTGGAACTTGG | 54 |
| CLUSTER-II (miR1861b- miR1861c) | CTGCTGGTTCTAACTTCGTCTTG | CCACCTTTGCATATTCACAGGC | 54 |
| CLUSTER-III (miR1861d-miR1861e) | TGCTGGTTCCAACTTCGTGTTG | CCACCTTTGCATATTCACAGACTC | 59 |
| CLUSTER-IV (miR1861f -miR1861g) | TGCTGGTTCTAACTTCGTCTTGC | CAAGTAGAGAGATCATTTGGGAAC | 54 |
| CLUSTER-V (miR1861h) | CATAAACGTTCTGCTGGTTCCAAC | GGTCATATTTACAAACGGATGCTGG | 54 |
| CLUSTER-VI (miR1861i) | AACGTTCTGCTGGTTCCAACTG | TGTCATATTCACCAGAGAAAATCACC | 54 |
| CLUSTER-VII (miR1861j- miR1861k) | GTTCTGCTGGTTCCAACTTCGTG | GGAGTAGAGAGATCATTCGGGAG | 62 |
| CLUSTER-VIII (miR1861l-miR1861m) | CATAAACGTTCCGCTGGTTCCAAC | GAGTAGAGAGATCATTCGGGAGC | 62 |
| CLUSTER-IX (miR1861n) | AACGTTCTGCTGGTTCCAACTGC | CAGTAGTAGGTCATATTCACAAACGG | 54 |
| **miRNA copy number primers** | | | |
| osa-miR1865 | CGAACTACTGCTAGTGATGATGATTC | GAGTCAAATTACAACTAGTGACTGCG | 54 |
| osa-miR1868 | GTAAAACACACTATTTGGCAGTTTGG | GCAAGAAACACACCGTTTAGAAGC | 50 |
| osa-miR1877 | GACATTCTCTCATTGCTTGCATGTC | CCTCATCATTCACATGTCATCTAAATGG | 50 |
| osa-miR1851 | CAGAGTGTCTTCGCCAAAATGCCATCC | TCCTCGTTGCCACCGCGTAGCC | 59 |
| osa-miR5153 | ATACGTGTTAGTTGGACTCACGTG | TACGTGTCAGTGGAATCCACATG | 57 |
| osa-miR5154 | TTAGACACCTCAGCGCCAAATAGC | AACTTAGACATCTCAGCGCCACG | 54 |
| osa-miR2092 | TTGATCTCCCAACTGAAGTCGGTG | ACACCTCTGCCAATGGAATGCTG | 57 |
| **qPCR primers** | | | |
| osa-miR 164e | TGGAGAAGCAGGGCACGTGAG | From the kit* | 59 |
| osa -miR169n | TAGCCAAGAATGACTTGCCTA | From the kit* | 59 |
| osa-miR169o | TAGCCAAGAATGACTTGCCTA | From the kit* | 59 |
| osa-miR171a | TGATTGAGCCGCGCCAATATC | From the kit* | 59 |
| osa-miR393a | TCCAAAGGGATCGCATTGATC | From the kit* | 59 |
| osa-miR394 | TTGGCATTCTGTCCACCTCC | From the kit* | 59 |
| osa-miR396c | TTCCACAGCTTTCTTGAACTT | From the kit* | 59 |
| osa-miR408 | CAGGGATGAGGCAGAGCATGG | From the kit* | 59 |
| osa-miR414 | TCATCCTCATCATCATCGTCC | From the kit* | 59 |
| osa-miR528 | TGGAAGGGGCATGCAGAGGAG | From the kit* | 59 |
| osa-miR1866 | TGGAAGGGGCATGCAGAGGAG | From the kit* | 59 |
| U6 | Provided with the kit^*^ | Provided with the kit^*^ | 59 |
| *Kit = Mir-X^TM^ miRNA First-Strand Synthesis kit (Clontech Laboratories, Inc.), Cat# 638313 | | | |

Table S2. Details of different types of primer sequences used in the present study
